# Comparison of Volatile and Nonvolatile Metabolites in Black Tea under Four Second-Drying Methods Using Widely Targeted Metabolomics

**DOI:** 10.3390/foods13010144

**Published:** 2023-12-31

**Authors:** Tianmeng Lan, Qingbin Zeng, Lin Chen, Zheng Tu, Yang Ye, Yueyun Liu, Weizhong He

**Affiliations:** 1 Tea Research Institute, Chinese Academy of Agricultural Sciences, Hangzhou 310008, China; 2 The University of Sydney Business School, University of Sydney, Camperdown, NSW 2006, Australia; 3 Department of Tea Science, College of Agriculture and Biotechnology, Zhejiang University, Hangzhou 310058, China; 4 Yibi Research Institute of Tea Industry, Yibi 644005, China; 5 Lishui Institute of Agricultural and Forestry Sciences, Lishui 323000, China

**Keywords:** second-drying, black tea, metabolomics, nonvolatile metabolites, volatile metabolites

## Abstract

Second-drying has an impact on the development of flavor and aroma in black tea. However, the effect of the shape changes of the tea leaves during second-drying on the quality of black tea has yet to be evaluated. In this study, GC-TOFMS and UPLC-HRMS identified 411 volatile metabolites and 253 nonvolatile metabolites. Additionally, 107 nonvolatile compounds and 21 different volatiles were screened. Significant alterations (*p* < 0.01) were found in 18 amino acid derivatives, 17 carbohydrates, 20 catechins, 19 flavonoids, 13 phenolic acids, and 4 organic acids. The content of certain amino acids and carbohydrates correlated with the shape of black tea. Furthermore, sweet aroma compound formation was facilitated by hot-air second-drying while the remaining second-drying approaches encouraged the formation of the fruity aroma compound. The results of the study provide a theoretical basis and technical instructions for the accurate and precise processing of premium black tea.

## 1. Introduction

Black tea is a highly consumed beverage worldwide [1]. As opposed to other tea types from China, black tea goes through full fermentation, which has a considerable effect on both the volatile and nonvolatile elements, resulting in a red tea soup. Typically, black tea has a rather sweet taste and aroma. Nevertheless, the utilization of distinct black tea processing technologies alters its flavor and fragrance [2]. Traditional black tea production involves several steps including withering, rolling, fermentation, first-drying, and second-drying.

The second-drying stage is crucial in black tea production, since it significantly affects both the aroma and flavor of the final product. Specifically, the application of high temperatures during this stage promotes the volatilization of low-boiling compounds such as the grass aroma. Additionally, this stage prolongs the duration and optimizes the conditions of the Maillard reaction, ultimately leading to an improved overall flavor and aroma of the tea [3]. Several studies have examined how a suitable temperature and duration of hot air second-drying affect the sensory characteristics of black tea [4]. The growing number of emerging drying technologies has led to diverse trends in black tea’s second-drying research including different types of thermal radiation, thermal convection, and heat conduction. These three methods have varying impacts on the quality of black tea formation [5]. Microwave drying, a thermal radiation method, rapidly elevates the temperature of tea leaves with high-frequency microwave oscillation. It proves effective in the second-drying of black tea by significantly augmenting the content of volatile compounds including aldehydes and ketones, ultimately enhancing the quality of black tea [6]. Heat pump drying is a form of heat convection that offers several benefits such as preserving the material quality, high efficiency, energy conservation, and pollutant-free operation. It efficiently mitigates the stale taste that arises from conventional high-temperature second-drying, and it can enhance the aromatic quality of black tea, fruits, and flowers [7]. Previous studies have either been limited to the effects of second-drying temperature or time, or have only compared the differences between different second-drying techniques. However, with the application of certain second-drying methods, the shape of tea leaves will also change [8,9]. Only a small number of studies have investigated the effects of different second-drying methods on the quality of green tea. These methods produce green tea with various shapes like straight, beaded, and curly. The physical shape changes of green tea may impact the degree of cell damage and heat, resulting in different green tea flavors [10]. However, the research on the shape and quality of black tea remains limited, and further examination is necessary to determine the effect of shape on black tea quality.

In order to understand the specific changes of the compounds of black tea with different drying methods, more advanced technologies are needed for detection and analysis. Time-of-flight mass spectrometry (TOFMS) is suitable for applications such as rapid gas chromatography separation and can compress peak times with sufficient sensitivity and speed [11]. In this study, complex volatile compounds in the samples were analyzed by gas chromatography-time of flight mass spectrometry (GC-TOFMS) combined with headspace solid phase microextraction (HS-SPME). Ultra-high performance liquid chromatography-high resolution mass spectrometry (UPLC-HRMS) can detect a large number of compounds with various organic functions, and has been fully applied in lipidomics, metabolomics, and proteomics research [12,13]. UPLC-HRMS was used for nontargeted metabolomics tests to deeply cover the metabolomics in this study. Through the above technology, the data of volatile metabolites and nonvolatile metabolites of the sample can be obtained more comprehensively.

Therefore, this study chose four widely used methods for the second-drying phase to create various shapes of black tea during processing. The objective was to examine the influence of these methods on the volatile and nonvolatile components of black tea to establish a theoretical foundation for enhancing the quality and visual appeal of black tea.

## 2. Materials and Methods

### 2.1. Chemicals

Mass spectrometry methanol, acetonitrile, and formic acid were purchased from Fisher Scientific, Fair Lawn, NJ, USA. Mass spectrometry ammonium bicarbonate was purchased from SIGMA-ALDRICH (Sigma-Aldrich Co., St. Louis, MO, USA). 2-Octanol (≥99.5%) was purchased from TCI Chemical Industry Development (Shanghai, China). Ultrapure water (18.2 M Ω·cm) was prepared using the Milli-Q purified water system (Merck KGaA, Darmstadt, Germany).

### 2.2. Sample Preparation

**Figure 1:**
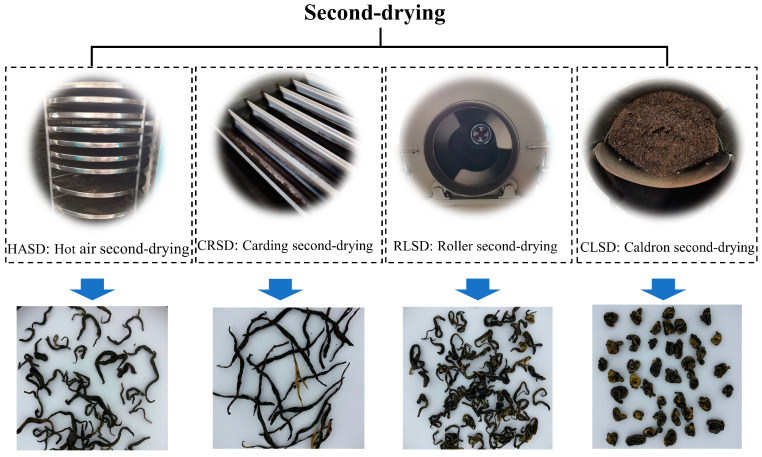
Four second-drying methods and the shape of the black tea.

The black-tea-making process is shown in Figure 1. Tea leaves (one bud and two leaves) from the cultivar “Fuxuan No. 9” were harvested in Yibin, Sichuan, China. The fresh leaves were withered, rolled, fermented, and first-dried. The first-dried tea leaves were placed in four machines for second-drying. We ensured that the surface temperature of each sample of tea reached a final temperature of 100 °C and the moisture content dropped below 6%. The specific parameters are as follows:

(1) Hot-air second-drying (HASD): The first-dried tea leaves were spread in a box-hot-air drying machine (JY-6CHZ-7B, Fujian Jiayou Machinery Co., Ltd., Quanzhou, China) and dried for 45 min.
(2) Roller second-drying (RLSD): The first-dried tea leaves were dried in a roller-frying pattern (6CCP-110; Fujian Jiayou Tea Patternry Intelligent Technologies Inc., Quanzhou, China) for about 35 min.
(3) Carding second-drying (CRSD): The first-dried tea leaves were dried in a carding-frying pattern (6CMD-6018, Zhejiang Lvfeng Patternry Co., Ltd., Quzhou, China) for about 20 min.
(4) Caldron second-drying (CLSD): The first-dried tea leaves were dried in a caldron-frying pattern (6CCGQ-50; Fujian Jiayou Tea Patternry Intelligent Technologies Inc., Quanzhou, China) for about 150 min.

After undergoing the second-drying process, Figure 1 showcases the shape of the ultimate black tea, with varying curl degrees of CLSD > RLSD > HASD > CRSD. CRSD denotes the straight shape of the tea, while HASD represents the natural form of the rolled tea after drying. RLSD corresponds to a slightly deeper curl, and CLSD indicates that the tea tightly wraps together, creating a granular shape after an extended period of second-drying processing. 

### 2.3. Analysis of Nonvolatile Compounds

A total of 200 mg (200 ± 2 mg) of each sample was weighed and placed in a 15 mL plugged centrifuge tube (Corning Incorporated, New York, NY, USA), and 5 mL of pre-heated methanol in a 70 °C water bath was added. After the tea powder was soaked, it was immediately moved into a 70 °C water bath for 10 min (the centrifuge tube was taken out in the middle and shaken for 2 min to make the tea powder fully suspended). After the centrifuge tube was cooled to room temperature, it was transferred to a centrifuge at 3500 r/min for 10 min, and the supernatant was transferred to a 10 mL volumetric flask. The residue was extracted with 5 mL of 70% methanol solution once, and the above operation was repeated. The combined extract was diluted to 10 mL, shaken well, and 2 mL supernatant was filtered with a 0.22 μm microporous membrane. The tea metabolite extract was transferred to the injection bottle for metabolomics analysis by liquid chromatography-high resolution orbitrap mass spectrometry.

The nontargeted metabolomics test was performed using Thermo Dionex Ultimate 3000TM ultra-high performance liquid chromatography (Thermo Fisher Scientific, San Jose, CA, USA) in series with Q-Exactive TM quadrupole-Orbitrap high-resolution mass spectrometry (Thermo Fisher Scientific, CA, USA), data acquisition software XCalibur 4.1 (Thermo Fisher Scientific, CA, USA). An Acquity UPLC HSS T3 column (2.1 × 100 mm, 1.8 μm, Waters Corporation, Milford, MA, USA) was used for separation. The mobile phase was 0.1% formic acid-water (phase A), and 0.1% formic acid-acetonitrile (phase B). Gradient elution conditions: 0–0.5 min, 5% B; 0.5–18 min, 5–40% B; 18–20 min, 40–90% B; 20–20.9 min, 90% B; 20.9–21 min, 10–95% A; 21–25 min, 5% B. The column oven was set to 40 °C, and the injection volume was 3 μL.

Mass spectrometric conditions [14]: The quadrupole-Orbitrap mass spectrometer was operated under identical ionization parameters with a heated electrospray ionization source, except for ionization voltage including sheath gas 45 arb, aux gas 10 arb, heater temperature 355 °C, capillary temperature 320 °C, and S-Lens RF level 55%. The metabolome extracts were profiled with full scan mode under a 35,000 FWHM resolution with AGC 1E6 and 200 ms max injection time. A 100~1000 *m*/*z* scan range was acquired. QC samples were repeatedly injected to acquire the Top 10 data-dependent MS2 spectra (full scan-ddMS2) for comprehensive metabolite structural annotation. A total of 17,500 FWHM resolution settings were used for full MS/MS data acquisition. Apex trigger, dynamic exclusion, and isotope exclusion were turned on, and precursor isolation window was set at 1.0 Da. Stepped normalized collision energy was employed for collision-induced disassociation of metabolite using ultra-pure nitrogen as fragmentation gas. All of the data were acquired as profile format. The full scan and data-dependent MS2 metabolic profiles (hydrophilic fraction) data were further processed with Compound Discoverer software (Thermo Scientific, San Jose, CA, USA) for comprehensive component extraction.

### 2.4. Analysis of Volatile Compounds

A 1000 ± 1 mg sample was taken into the 20 mL headspace bottle, 5 mL saturated sodium chloride was added, and 10 μL of 2-Octanol (10 mg/L stock in dH2O) was added as the internal standard; All samples were analyzed by a gas chromatograph system coupled with a spectrometer (GC-MS).

In the SPME cycle of the PAL rail system, the incubation temperature is 60 °C; preheat time is 15 min; incubation time is 30 min; desorption time is 4 min. GC-MS analysis was performed using an Agilent 7890 gas chromatograph system coupled with a 5977B mass spectrometer. The system utilized a DB-Wax. Injected in Splitless Mode. Helium was used as the carrier gas, the front inlet purge flow was 3 mL min−1, and the gas flow rate through the column was 1 mL/min. The initial temperature was kept at 40 °C for 4 min, then raised to 245 °C at a rate of 5 °C/min, and kept for 5 min. The injection, transfer line, ion source, and quad temperatures were 250, 250, 230, and 150 °C, respectively. The energy was −70 eV in electron impact mode. The mass spectrometry data were acquired in scan mode with the *m*/*z* range of 20–400, a solvent delay of 0 min.

Chroma TOF 4.3X software from LECO Corporation (St. Joseph, MI, USA) and the Nist database were used for exacting the raw peaks, the data baseline filtering and calibration of the baseline, peak alignment, deconvolution analysis, peak identification, integration, and spectrum match of the peak area [15].

### 2.5. Statistical Analysis

PCA, PLS-DA, and OPLSD-DA of the volatile and nonvolatile compounds were performed using SIMCA 14.1 software (Umetrics, Umea, Sweden). Differences were based on one-way analysis of variance (ANOVA) using the SPSS statistics 25 software (SPSS Inc., Chicago, IL, USA). Figures were drawn by Origin 2022 (OriginLab Corporation, Northampton, MA, USA) and an online website https://www.omicstudio.cn/tool (accessed on 1 November 2023).

## 3. Results

### 3.1. Changes of Nonvolatile Compounds in Different Second-Drying Methods

**Figure 2:**
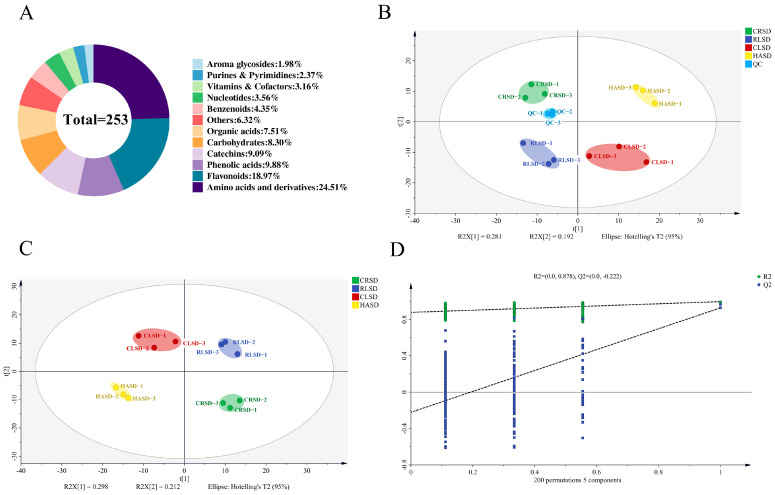
Nonvolatile compound composition and multivariate analysis. (**A**) Number of nonvolatile compounds and percentage of each subcategory. (**B**) PCA score scatter plot composed of all nonvolatile metabolites. (**C**) PLS-DA scores plot. (**D**) Cross-validation results with 200 times of calculations by using a permutation test.

A total of 253 nonvolatile compounds were identified in this study (Table S2) including 62 amino acids, 48 flavonoids, 25 phenolic acids, 23 catechins, 21 carbohydrates, 19 organic acids 11 benzenoids, 9 nucleotides, 8 vitamin minerals, 5 aroma glycosides, and 16 other compounds. (Figure 2A). To evaluate the within-group reproducibility and the between-group variability, we conducted an unsupervised PCA modeling analysis on the 253 nonvolatile compounds above-mentioned. In the PCA plot (Figure 2B), the quality control samples (QC) were tightly clustered together, indicating that the performance of the experimental platform maintained stability during analysis and that the quality of the test data were reliable. According to the PCA results, the four second-drying samples were evenly spread out across the four quadrants of the coordinate axes. This suggests that there was variability among the samples, but none of them had exceptionally high variability. Additionally, the individual samples were grouped, indicating good intra-group reproducibility and meeting the requirements for further analysis.

To preliminarily screen out the differential nonvolatile metabolites, multivariate analysis with PLS-DA and univariate analysis with one-way ANOVA were conducted. The PLS-DA score plot (Figure 2C) showed a significant difference between the four samples, indicating varying contents of nonvolatile metabolites resulting from different second-drying methods. After 200 permutations (Figure 2D), the value and intercept of R2 were 0.996 and 0.878, respectively, and the value and intercept of Q2 were 0.928 and −0.222, respectively, which indicates that the model had good interpretability and credibility. Based on this, the VIP value of each compound was calculated, and the *p*-value of each compound was calculated using the single factor analysis in SPSS. A total of 107 differential metabolites with VIP > 1 and *p* < 0.01 were screened.

#### 3.1.1. Amino Acids and Derivatives

Amino acids make up approximately 1–4% of dried tea [16] and are essential in imparting a fresh and invigorating taste in tea soup [17]. The figure reveals that under CRSD and RLSD conditions, the levels of most amino acids significantly rose (*p* < 0.01), followed by HASD, while the content of CLSD was the least. From the shape of Figure 1, it is evident that this trend correlates with the form of black tea. For instance, the straight CRSD results in a larger heated surface area led to a rapid and uniform temperature rise during tea processing. Conversely, the rolled and granular CLSD hindered quick heat transfer to the inside due to continuous movement, resulting in slower and uneven heating. Subsequently, these processes could potentially impact the hydrolysis of amino acid precursors, causing a decrease in amino acid content. Furthermore, CRSD and RLSD exhibited the highest levels of L-glutamine, L-lysine, and L-phenylalanine, with a trend of CRSD > RLSD > CLSD > HASD. When solely analyzing the heat conduction mode, there was a negative correlation between the curl degree of the black tea shape and the quantities of these amino acids. Investigating the reason behind this trend of amino acids would be valuable. The levels of N-alpha-acetyllysine, N-acetylthreonine, pyroglutamic acid, acetamidobutanoic acid, and N-acetyl-L-aspartic acid were greatest in HASD, which differed significantly from the three other methods of heat conduction (*p* < 0.01). However, there was no notable correlation between these components and the shape of the tea. It should be highlighted that the gamma-aminobutyric acid content was highest in RLSD, followed by CRSD. It is reported that this substance has the effect of reducing anxiety [18]. At present, tea processing mainly increases its content by anaerobic treatment [19,20], which provides a new idea for different drying methods.

#### 3.1.2. Carbohydrates

**Figure 3:**
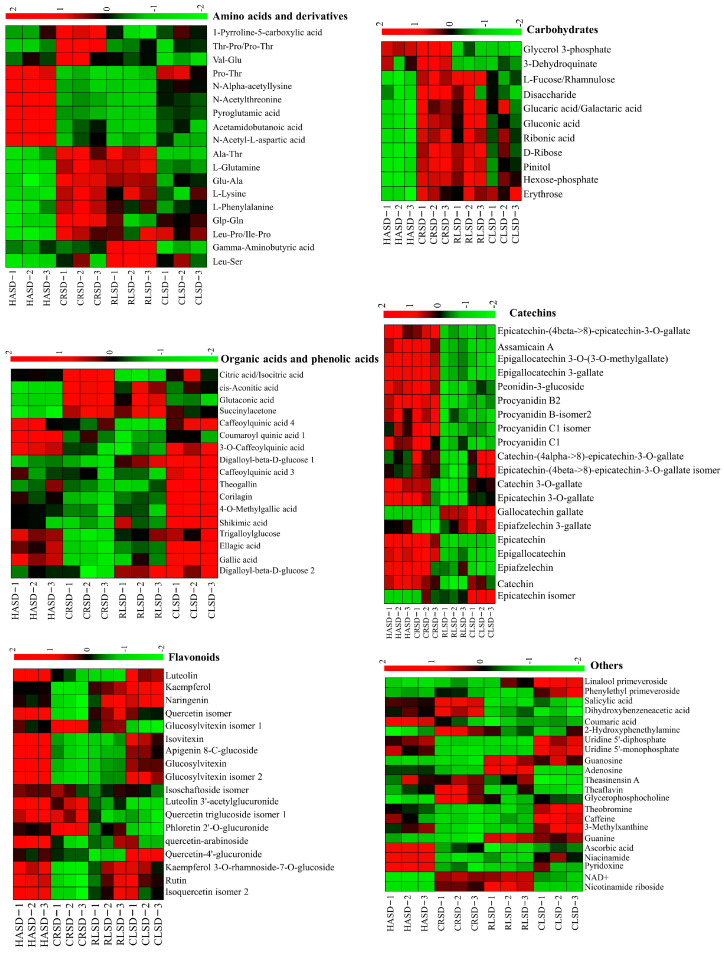
Heat maps of the levels of the 107 differential nonvolatile metabolites during different second-drying methods. HASD, hot air second-drying; CRSD, carding second-drying; RLSD, roller second-drying; CLSD, caldron second-drying.

Carbohydrates consist of monosaccharides, polysaccharides, disaccharides, sugar alcohols, glycogen, and other compounds [21]. According to Figure 3, the carbohydrate levels showed significant increases under CRSD conditions, followed by RLSD and CLSD, with the least amount observed under HASD conditions. Therefore, our speculation is that tea curl increases can cause an uneven surface temperature of the tea and decrease the hydrolysis of carbohydrate precursors, ultimately affecting carbohydrate formation. It is notable that despite not having the greatest degree of bending, HASD had the lowest carbohydrate content, possibly due to differences in heat transfer mode and time. Further investigation is necessary to determine the exact reasons. Sugars are crucial metabolites in black tea’s sweetness. Our findings demonstrate a significant increase in carbohydrates in tea under heat conduction mode. The carbohydrates included monosaccharides (D-ribose, hexose-phosphate, erythrose, etc.), disaccharides, pinitol, and other sugar alcohols. Therefore, it is hypothesized that the use of heat conduction mode in the second-drying method with a lower-bending degree can potentially elevate the carbohydrate content, ultimately resulting in a sweeter taste of the black tea soup.

#### 3.1.3. Organic Acids and Phenolic Acids

Organic acids have been found to positively inhibit bitterness and sourness [22]. In our study, the content of organic acids in HASD was the lowest, while it was significantly higher in CRSD. Our study suggests that the CRSD condition may be particularly effective in enhancing the organic acids content. Additionally, the content of phenolic acids increased significantly (*p* < 0.01) under CLSD conditions (CLSD > HASD > RLSD > CRSD), positively correlating with the heating time. This finding indicates that excessively long heating times may lead to increased phenolic acid content. Additionally, the degree of curling in black tea is correlated with this trend. According to research by Asamenew [23], the roasting process substantially decreases the amount of caffeoylquinic acid. However, our study found that under CLSD and HASD conditions, the levels of caffeoylquinic acid 3, caffeoylquinic acid 4, and 3-O-caffeoylquinic acid significantly increased. This suggests that the initial roasting temperature should also be taken into account. Phenolic acids may contribute to the bitterness and astringency of tea, as noted by Frank [24]. To enhance the bitter taste of tea during production and processing, high temperature and short-time drying methods can be utilized to induce a strong redox and hydrolysis reaction, promoting the conversion of phenolic acids. Our findings confirm this hypothesis, particularly under CRSD conditions, in which a notable rise in phenolic acids could be linked to the high temperature and brief duration of the drying process. These observations enhance our knowledge of the organic and phenolic acid variations associated with varying drying techniques and offer valuable theoretical support for refining the production process of black tea.

#### 3.1.4. Catechins and Catechin Derivates

Catechins are essential components that determine the taste concentration and convergence in black tea. The suitable distribution ratio and overall catechin amount can regulate the taste of the tea infusion. According to Figure 3, the majority of catechin content was significantly higher under HASD and CRSD conditions compared to RLSD and CLSD. However, catechin-(4alpha->8)-epicatechin-3-O-gallate, epicatechin-(4beta->8)-epicatechin-3-O-gallate isomer, gallocatechin gallate, epiafzelechin 3-gallate, and epicatechin isomer exhibited the opposite trend. CRSD had the shortest drying time, leading to insufficient degradation and oxidation of many catechin dimers. The drying time of HASD was moderate, but no mechanical friction with the pot caused further cell breakage, so the content of catechin dimers in CRSD and HASD was significantly increased. The tea leaves during RLSD and CLSD treatment were in contact with the high-temperature pot for a long time, and a series of oxidative degradation reactions occurred, resulting in a significant decrease in catechin dimers.

#### 3.1.5. Flavonoids

In our results, the selected differential flavonoids included 4 flavonoids and 15 flavonoid glycosides. In the HASD method, the total content of these compounds was significantly higher than the three other heat conduction methods. It has been reported that flavonoid glycosides are significantly hydrolyzed during the rolling process of black tea [25]. In this study, in the early stage of drying, the temperature rose slowly, and the tea continuously collided with the pot, which further increased the cell breakage rate. This mechanical movement process is similar to the rolling process, which significantly reduced most of the flavonoid glycosides. In addition, the contents of three nonflavonoid glycosides, kaempferol, naringenin, and quercetin isomer, were relatively high in RLSD and CLSD. The RLSD and CLSD methods made black tea curly and granular, making it difficult to contact the high-temperature pot inside the tea, which was conducive to longer enzymatic hydrolysis to form flavonoids. In addition, the changes in the content of other flavonoids under different drying times also changed differently. A preliminary view, compared with the thermal cracking reaction, is that enzymatic hydrolysis may be the main factor in the significant reduction in flavonoid glycosides.

#### 3.1.6. Other Compounds

In this part, the compounds that have an important correlation with the quality of black tea were preliminarily analyzed. Theaflavins are important compounds that affect the brightness, taste intensity, and freshness of the infusion [26]. As shown in Figure 3, the content of theaflavins in CRSD was significantly higher than that in other drying methods. This result indicates that the tea infusion after CRSD treatment may be brighter. In addition, caffeine, as one of the bitter components in tea, should also be noted. In comparison, the content of theobromine and caffeine in CLSD was significantly higher than that in the other groups, followed by HASD, RLSD, and CRSD, which means that the content of bitter compounds can be improved by different drying methods.

The heating time of tea varies based on the number of materials used and the structure of the second-drying equipment. Among them, the heating time of CLSD was the longest, the low-temperature stage lasted longer, and the enzyme activity was also maintained longer. This situation produces the enzymatic hydrolysis reaction to form more flavonoids. On the other hand, in addition to the known effects of temperature and time on nonvolatile substances in black tea, through our experimental results, we also found that the shape of the tea affected the temperature transfer efficiency from the surface to the interior of the tea, thus affecting the formation of some nonvolatile metabolites. In the experiment, the characteristics of CLSD led to a relatively slow heating rate of the tea and a longer low-temperature stage, which provided conditions for the continuous activity of the enzyme. In contrast, the characteristics of other second-drying methods made the tea temperature rise rapidly, resulting in a short low-temperature stage. This finding provides a new perspective for understanding the effects of different second-drying methods on the formation of bioactive substances in black tea. In addition, our study also shows that the shape of the tea affects the temperature transmission efficiency. Different shapes may lead to inconsistent temperature changes on the surface and inside of the tea leaves, thus affecting the formation of organic matter. Specifically, some amino acids, carbohydrates, and phenolic acids may be different in different shapes of tea. This finding is of practical importance for optimizing the production process of black tea and increasing the yield of organic matter.

During the drying process of black tea, various compounds are produced and degraded primarily due to thermal-driven processing. Significant differences were observed in this experiment among the different second-drying methods. For instance, CRSD and RLSD had shorter drying times and higher temperatures, resulting in higher amino acid contents. During fermentation, enzyme-catalyzed reactions have a limited effect on amino acids [27]. However, high temperature may promote protein hydrolysis [28]. Therefore, we hypothesized that the differences in amino acid contents could be due to the varying drying temperature. Additionally, certain enzymes were not denatured and inactivated during the early stages of drying, which suggests that enzyme-catalyzed reactions may also be involved in the production and degradation of compounds. In this study, it was found that theaflavins were highest in CRSD and lowest in CLSD. The process of the enzyme-catalyzed reaction for theaflavins has been thoroughly researched: catechins are oxidized to o-quinone, which then couples with residual catechins to produce theaflavins. This is followed by the formation of thearubigins [29,30]. It is hypothesized that the low drying temperature and long drying time of CLSD result in slow enzyme-catalyzed reactions, leading to further oxidation of theaflavins and a subsequent decrease in their content (refer to Figure 3). Overall, different second-drying methods can impact the shapes of tea leaves, which in turn affects the temperature of the surface of the tea leaves and the degree of enzyme-catalyzed reaction. However, the thermal-driven reaction remains dominant. The interaction between these two reactions can impact the formation and degradation of metabolites.

### 3.2. Changes of Volatile Compounds in Different Second-Drying Methods

#### 3.2.1. Composition of Volatile Compounds

**Figure 4:**
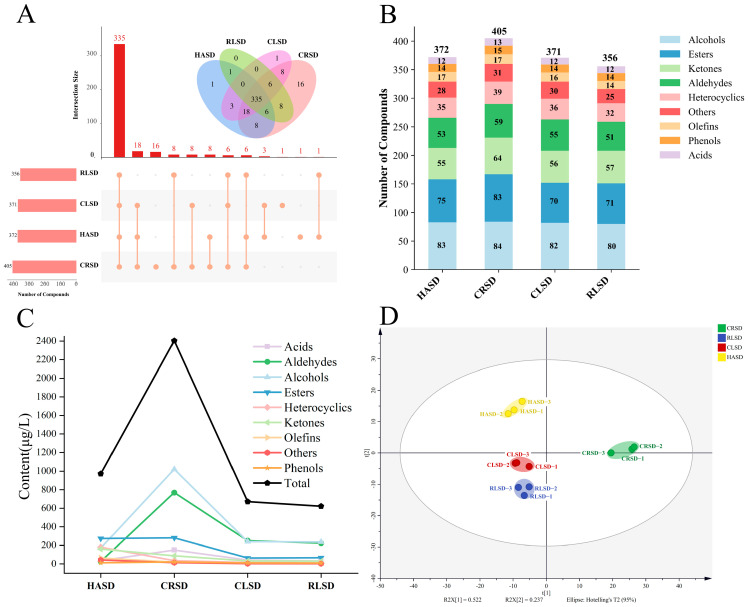
Comparison of the composition of volatile compounds and multivariate analysis. (**A**) Upset plot of volatile compounds for four second-drying methods. (**B**) Histogram of the number of subclasses of volatile compounds for four second-drying methods. (**C**) Variations in the concentrations of volatile compounds for four second-drying methods. (**D**) PCA score scatter plot composed of all volatile metabolites.

To gain a better understanding of how metabolites change during second-drying, we used nontargeting GC-TOMFS technology to identify volatile metabolites in the samples (Table S1). HASD, CRSD, CLSD, and RLSD contained 372, 405, 371, and 356 volatile metabolites, respectively, and these compounds included alcohols, esters, ketones, aldehydes, heterocycles, phenols, acids, olefins, and others (Figure 4B). Figure 4A displays upset diagrams of volatile metabolites in four different second-drying methods. The purpose of these diagrams is to present complex Venn diagrams in a more intuitive way. The solid points and lines that connect represent intersections between different sets, with the histogram above displaying the number of each intersection. From the figure, it is evident that the top three volatile compounds were ranked 335, 18, and 16, respectively. A total of 335 volatile compounds were found in all four groups of second-drying methods, making up the majority of all volatile compounds. This suggests that the black tea aroma substances in all four groups of samples were similar. Only 18 volatile compounds were absent in RLSD, while CRSD had 16 unique volatile compounds. Overall, CRSD ranked highest in terms of the volatile compounds it covered, while the other three second-drying methods shared different volatile compounds. Notably, CRSD did not contain maltol, a volatile compound with a caramel flavor [31], which was present in both HASD and CLSD. This outcome holds great significance in comprehending the impact of varied second-drying techniques on the volatile metabolites of black tea. The analysis of Figure 4C further examined the total volatile compounds and concentrations of each compound type. CRSD exhibited the highest total compound concentrations, particularly in alcohols and aldehydes, which were significantly greater than in the other samples. Furthermore, the esters in CLSD and RLSD were significantly lower than those found in HASD and CRSD, possibly attributed to the shape of the tea leaves. During the continuous curling process of tea leaves, there is minimal contact between the internal tea leaves and the pot [11], which poses difficulties in transferring heat, thereby resulting in challenges with the hydrolyzing and synthesizing of numerous aroma substances.

PCA was still utilized to perform a preliminary analysis of sample repeatability and variability (Figure 4D). Four groups of sample replicates were all well-clustered together, suggesting that the experiments were well-reproducible. In PC1, the CRSD sample was notably separated from the other samples, suggesting increased variability in volatiles, which may impact the creation of the black tea aroma.

#### 3.2.2. Changes of Core Differential Volatile Compounds

**Figure 5:**
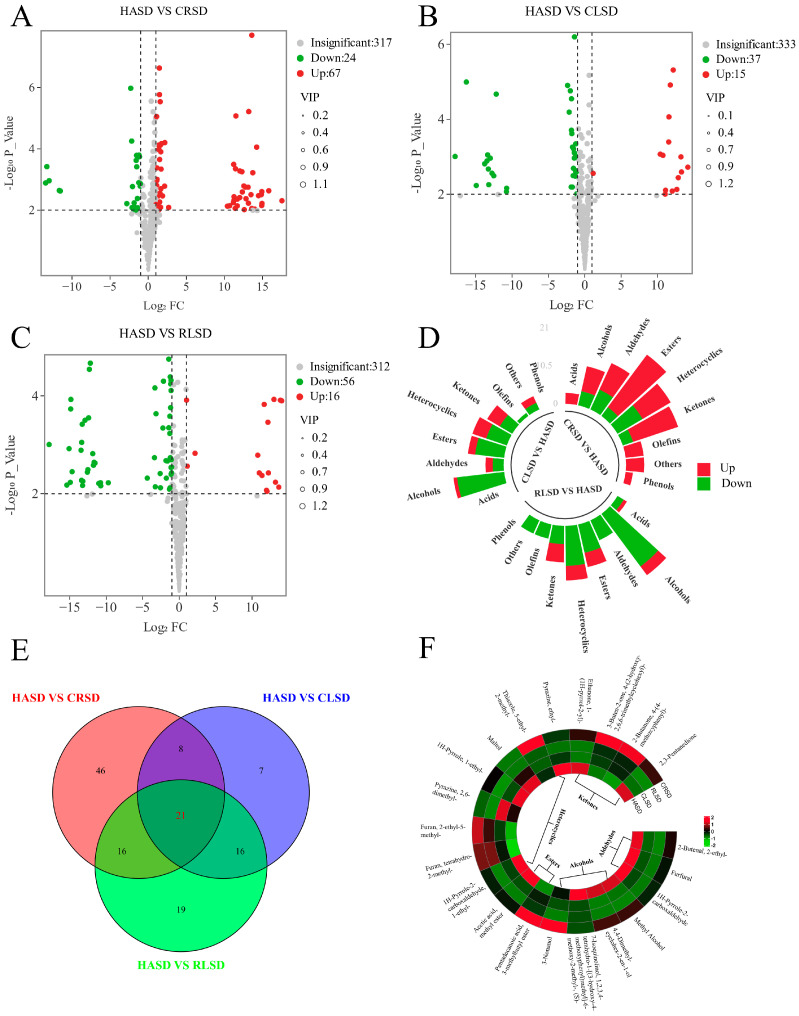
Screening and analysis of differential compounds. (**A**–**C**) Volcano plots of HASD vs. CRSD, HASD vs. CLSD, and HASD vs. RLSD, respectively. Screening conditions, VIP > 1, *p* < 0.01, and fold change < 0.5 or fold change > 2. (**D**) Cyclic histograms of subclasses of differential compounds. (**E**) Venn diagram of the differential nonvolatile metabolites of HASD vs. CRSD, HASD vs. CLSD, and HASD vs. RLSD. (**F**) Circumferential heat map of 21 core differential metabolite levels.

Hot-air drying is the primary method for drying black tea in large-scale production. Our thorough analysis examined the impacts of various processing methods on volatile compounds, focusing on the core group HASD. We conducted a two-by-two comparison between HASD and the remainder of the group. To obtain the VIP value, we utilized the triple screening criteria of VIP value, *p*-value, and FC, OPLS-DA model. Additionally, the Student’s *t*-test was utilized to obtain the *p*-value. A total of 133 metabolites showed differential expression based on the screening criteria of VIP > 1, *p*-value < 0.01, and a fold change >2 or <0.5. Figure 5A shows 91 metabolites that were differentially expressed between HASD and CRSD, with 67 upregulated and 24 downregulated. In Figure 5B, there were 52 differential metabolites in HASD and CLSD, of which 15 were upregulated and 37 were downregulated. Finally, Figure 5C demonstrates 72 differential metabolites in HASD and RLSD, with 16 upregulated and 56 downregulated.

We analyzed the quantity and types of differentiated compounds in each of the aforementioned groups (Figure 5D). Our findings indicate a marked increase in the number of upregulated compounds across all subclasses in CRSD, in contrast to HASD. Additionally, we noted a substantial increase in downregulated compounds in both CLSD and RLSD. These results suggest that diverse drying procedures may influence the overall amount of volatile compounds, rather than specific types alone. The extended drying time causes the volatilization of many alcohols, leading to a decrease in the alcohol content in CLSD, while elevated temperatures and rapid drying can facilitate esterification reactions [28], resulting in increased CRSD esters. Using the Venn diagram illustrated in Figure 5E, we identified 21 key compounds that have the potential to differentiate hot-air drying from heat conduction drying. Among these compounds were three aldehydes, four alcohols, two esters, eight heterocycles, and four ketones. As shown in Figure 5F, the concentrations of furfural, 1H-pyrrole-2-carboxaldehyde, pyrazine,2,6-dimethyl-, pyrazine, ethyl-, and maltol, which impart a roasted and sweet fragrance, decreased after heat conduction drying. On the other hand, the levels of 3-nonanol, pentadecanoic acid, 3-methylbutyl ester, furan, 2-ethyl-5-methyl-, and 2-butanone, 4-(p-methoxyphenyl)-, which contribute to a fruity and fresh aroma, increased. Therefore, it can be surmised that hot-air drying promotes the development of a sweet aroma, whereas heat conduction drying facilitates the formation of a fruit odor. This offers insightful concepts for manufacturing black tea with varying aroma profiles.

During the drying process, volatile compounds undergo changes due to heat. High temperatures promote the production of specific compounds such as 5- and 6-methyl-2-ethylpyrazine, 2-ethyl-3,5-dimethylpyrazine, 3,5-diethyl-2-methylpyrazine, 5-hydroxymethylfurfural, and furfural, which create bakery and caramelized aromas [32,33]. Some of the volatiles produced by Maillard reactions include pyrazines and pyrroles. In this study, the levels of pyrazines and pyrroles in HASD were high, and we hypothesized that the appropriate time and temperature are crucial factors. It is possible that the different drying methods used may have contributed to these results, which require further exploration. Wang et al. found that a brief drying period could result in a floral flavor [4], but the tea temperature was not high during this process. In our study, particularly in CLSD, we observed more prominent floral and fruity aromas. We hypothesize that this is due to the relatively low temperature and short duration required to achieve the desired moisture content of black tea. These aroma substances with low boiling points are less likely to evaporate, which helps to preserve some of the floral and fruity aromas.

## 4. Conclusions

Here, we conducted an extensive targeted metabolomics analysis to study the effects of second-drying on both volatile and nonvolatile metabolites in black tea. Our analysis revealed 253 nonvolatile and 411 volatile metabolites. Among those, we identified 107 differential nonvolatile metabolites and 21 core differential volatile metabolites. Our findings suggest that various heat transfer methods significantly impact the volatile compounds present in black tea. Heat conduction second-drying methods including CRSD, CLSD, and RLSD resulted in an increase in fruit-aroma compounds, while HASD led to higher levels of roasted and sweet-smelling compounds like furfural. On the other hand, HASD showed an increase in flavonoids and catechins in nonvolatile compounds, whereas heat transfer promoted the accumulation of amino acids and carbohydrates. These changes in key metabolites can function as effective indicators for achieving optimal and standardized processing of high-quality black tea. Additionally, as the curl of the black tea deepens, certain amino acids and carbohydrates decrease while some flavonoids increase. The alteration in the black tea shape and the duration of the second-drying time can also impact the development of alcohols, esters, aldehydes, and other compounds. This study presents a theoretical framework and technical guidance for the accurate directional processing of black tea with various shapes and flavors.

## Data Availability

Data is contained within the article or supplementary material.

## Supplementary Materials

The following supporting information can be downloaded at: https://www.mdpi.com/article/10.3390/foods13010144/s1, Table S1: List of the volatile compounds in the black tea samples; Table S2: List of the nonvolatile compounds in the black tea samples.
