# Supplementary material for: Comparison of Volatile and Nonvolatile Metabolites in Black Tea under Four Second-Drying Methods Using Widely Targeted Metabolomics
Source: Foods. 2023 Dec 31;13(1):144. doi: 10.3390/foods13010144 (PMC10778963; doi:10.3390/foods13010144)
Supplement: Supplementary file 1 [file foods-13-00144-s001.zip › foods-2786241-supplementary.pdf]

Table S1. List of the volatile compounds in the black tea samples.

No.	Compounds	Peak area											
		CRSD-1	CRSD-2	CRSD-3	RLSD-1	RLSD-2	RLSD-3	CLSD-1	CLSD-2	CLSD-3	HASD-1	HASD-2	HASD-3
1	1H-Tetrazole-1,5-diamine	27503.435	24347.0625	21190.69	19034.734	19799.05	24067.136	26489.46	20978.231	23941.486	29260.407	21902.276	31129.323
2	Methanethiol	210508.243	197152.054	183754.941	156663.709	171216.363	156751.398	167006.688	158576.02	157614.181	192323.355	165278.11	199960.56
3	Propanal	713387.578	729220.745	673157.279	615565.314	718494.127	627300.107	551846.82	559049.206	587454.168	592762.661	571633.454	608785.45
4	Sesquicineole	125874.808	111385.384	96895.96	120075.468	107006.007	90042.642	42783.784	54568.061	66352.338	83205.265	76977.544	80091.4045
5	Propanal, 2-methyl-	11380372.3	11400767.3	11552686.8	9840504.62	10612909.2	10242418	13269087.7	13559013.2	13658328.4	15701097.6	14167184.5	14752583.3
6	Acetic acid, methyl ester	53787.504	50728.714	58490.812	nd	nd	nd	49248.585	51597.25	53945.915	271623.905	265277.266	261723.575
7	2-Propenal	554161.145	545738.641	552352.643	461564.942	500741.082	478407.739	429780.219	453700.906	442223.052	427330.056	361386.823	397096.7
8	Furan, tetrahydro-2-methyl-	83821.103	84181.0235	84540.944	157227.288	154228.24	151229.192	86941.378	91726.692	82156.064	nd	nd	nd
9	Butanal	445357.543	418839.826	412428.3	256282.881	324098.54	263653.3	357298.081	342338.169	345146.387	532941.091	516248.066	626287.996
10	4-Hydroxy-2,6,6-trimethylcyclohex-1-enecarbaldehyde	123843.745	125021.49	116890.588	140811.045	110898.306	80985.567	107559.477	122052.595	123779.345	92104.826	96791.74	119641.654
11	Ethyl Acetate	126548.395	106673.661	125771.041	262799.69	236983.869	250882.541	126061.878	143753.477	141685.909	115689.385	116908.399	113891.936
12	Phthalic acid, heptyl tridec-2-yn-1-yl ester	106407.033	99576.845	92746.657	215453.873	173329.4655	131205.058	179156.3795	165997.094	192315.665	110203.879	182110.924	146157.4015
13	Methyl Alcohol	934708.632	931154.037	882425.581	442453.75	454405.539	442772.05	601040.948	599697.487	563451.877	2140830.34	2131946.63	2024102.07
14	2-Butanone	469202.143	450538.803	484605.326	427081.086	453317.368	424805.956	506151.443	489814.258	484276.729	679068.264	638059.107	711152.455
15	cis-2-(2-Pentenyl)furan	143837.491	167940.085	119734.897	nd	nd	nd	nd	nd	nd	nd	nd	nd
16	1H-Indole, 1-methyl-2-phenyl-	21079.613	19680.919	18282.225	nd	nd	nd	17283.952	14557.484	15920.718	13781.334	18048.6075	15914.97075
17	Butanal, 3-methyl-	22251899	21635988	21708437.7	17556604.8	20839074.3	18972296.9	21935159.8	21025218.1	22706046	21970376.4	19555740.1	21616891.1
18	Ethanol	2990375.82	2721955.53	2746319.45	3029855.99	2767202.7	2791225.35	2824926.06	2731693.44	2729203.63	2569954.22	2456238.87	2328336.93
19	Furan, 2-ethyl-	5778769.44	6658343.84	6006364.6	3457107.37	3923089.51	4634338.06	4795217.36	3962742.35	3735322.5	3931563.54	3512349	3122369.11
20	1,3-Octadiene	42254.637	45218.852	39290.422	nd	nd	nd	55068.999	47416.672	51242.8355	31073.873	35575.848	33324.8605

21	Sulfurous acid, 2-pentyl pentyl ester	60797.649	55721.517	57851.055	57843.869	66236.789	68049.837	67669.038	55994.396	60482.969	52687.711	41250.416	45039.395
22	3-Ethyl-3-methyl-2-pentanol	62157.074	57525.283	60479.695	52349.8265	57969.112	46730.541	63567.891	65604.089	62304.218	65770.324	50448.577	59803.201
23	1,3-Cyclopentadiene, 5-(1,1-dimethylethyl)-	187974.869	232578.656	210276.7625	106246.417	132688.559	117580.98	140777.397	127467.416	121011.578	82294.48	61006.652	71650.566
24	Oxalic acid, monoamide, n-propyl, heptyl ester	753886.401	807466.826	677011.238	600413.938	625924.855	641525.91	591700.04	614603.874	631243.269	670020.748	649368.453	681315.979
25	Pentanal	11948983.3	13155149.6	9999693.63	9849302.89	10196384.4	10907685.8	10456255.1	9856763.35	10148610.7	6321184.15	5208658.63	6220399.78
26	Hexyl octyl ether	894571.658	1075935.44	713207.876	653119.713	630059.92	650426.945	371587.826	447585.929	409586.8775	515737.127	531909.718	720657.519
27	Butane, 1-methoxy-3-methyl-	577313.692	730794.167	662947.387	194731.687	210304.895	221467.736	141406.825	162680.1915	183953.558	563637.999	565594.377	550754.06
28	1,5-Heptadiene, 2,6-dimethyl-	49350.598	58146.378	53939.832	nd	nd	nd	nd	nd	nd	47723.753	52942.2295	58160.706
29	2H-Pyran, 3,4-dihydro-6-methyl-	42990.075	43181.93	44685.374	34449.045	40716.43	37100.308	35313.251	45586.067	42711.049	66646.378	65543.854	61151.918
30	3-Pentanone, 2-methyl-	40875.41	42808.875	40591.982	53170.888	55077.717	57547.371	40240.789	35920.957	36441.155	46786.717	41530.33	44158.5235
31	Pentanal, 2-methyl-	193926.885	217889.383	198476.785	139832.459	149767.086	143171.787	225286.233	235318.804	230282.292	404902.895	337870.926	347147.069
32	Acetic acid	1697201.71	1902598.04	1865848.72	1256227.79	1528836.7	1392532.245	1683367.83	1670532.12	1657696.41	1961014.68	1323134.89	1642074.785
33	1-Penten-3-one	3242102.1	3183747.12	3002072.47	2708787.73	2934118.6	2762353.48	2354690.91	2358891.53	2242006.51	2111320.23	1850040.68	2097836.4
34	Phthalic acid, butyl hex-3-yl ester	2494528	2653863.07	2574195.535	2318525.18	1761924.29	1913391.15	2424302.66	2133529.21	1842755.76	1696869.19	1688838.75	1734140.32
35	2,3-Pentanedione	218687.759	238093.278	227142.7	177939.679	194783.708	204337.756	221170.06	225266.567	235426.433	489154.057	476862.159	438089.381
36	Sulfurous acid, hexyl nonyl ester	174532.907	208047.887	173293.944	nd	nd	nd	106183.3185	113406.52	98960.117	68737.451	47379.8	58058.6255
37	Pyridine, 3-(trifluoromethyl)-	182716.272	184691.311	188023.598	159830.121	183429.209	176426.14	249507.802	252297.051	254825.014	219967.023	193866.087	178460.257
38	1-Penten-3-one, 2-methyl-	840029.016	984111.537	912070.2765	757266.876	796789.897	702335.854	869487.686	804370.677	856008.729	631639.132	494072.169	562855.6505
39	Acetic acid, butyl ester	57900.151	54611.529	57073.825	45759.111	56674.842	57368.898	48372.89	51364.001	44584.976	61412.327	50933.489	51395.2
40	Hexanal	20902832.7	19714244.3	19161996.5	16441617.7	17515548.5	18457180	14772948.7	14699322.3	14118809.8	16086484	14470785.9	13479228.4
41	Eugenol	1207281.88	1007566.97	1107424.425	846304.4485	699577.2178	552849.987	458520.6015	451930.69	465110.513	553589.679	494677.818	524133.7485
42	Pentadecanoic acid, 3-methylbutyl ester	283085.041	319513.231	243317.104	117054.3835	99587.859	134520.908	179415.553	166800.1995	154184.846	nd	nd	nd
43	Sulfurous acid, butyl nonyl ester	69810.413	82509.758	76160.0855	27798.4	23938.902	31657.898	45045.4685	50250.044	39840.893	28654.909	18284.858	23469.8835
44	2-Propyl-1-pentanol	7928024.04	8103373.16	8015698.6	10838394.2	9499747.25	8552775.08	9918080.87	7321761.52	8619921.195	7825067.04	7140155.53	8757714.17

45	2,4,4-Trimethyl-3-(3-methylbutyl)cyclohex-2-enone	87561.773	98193.421	92877.597	87653.163	93691.8145	99730.466	119292.005	112623.863	115957.934	94284.48	93135.425	71527.313
46	Trimethylamine, 1-cyclohexyl-	69471.27925	78028.0305	60914.528	133080.344	103096.458	118088.401	nd	nd	nd	68113.707	56725.221	62419.464
47	Pentanoic acid, 2-methyl-, methyl ester	5292547.848	5929763.125	4655332.57	3485551.31	3215784.02	3422599.35	4010424.35	3592865.54	3489712.03	3391206.27	2994728.43	3192967.35
48	Di-epi-1,10-cubenol	21961.125	24602.93	19319.32	nd	nd	nd	nd	nd	nd	nd	nd	nd
49	Carbonic acid, 2-ethylhexyl nonyl ester	1070055.78	969958.933	1020007.357	216650.282	207747.12	225553.444	444617.645	400567.1935	356516.742	258422.208	191430.516	224926.362
50	2,7-Octadien-1-ol	825563.2863	924557.0175	726569.555	653573.479	801024.352	718361.279	890730.659	915952.238	906548.983	1038119.02	892338.154	965228.587
51	1-Penten-3-ol	30309400.5	30388613.7	32280704	12575965.4	13064697	12925059.7	11886976.2	13205041.6	12788792	31044196.7	30101127.7	28229797.2
52	2-Propenoic acid, butyl ester	1181760.3	1347016.92	1071836.74	665025.868	626797.874	703253.862	611010.067	691771.759	594922.174	1184245.11	1002721.54	1123852.39
53	1H-Pyrrole-2,5-dione, 3-ethyl-4-methyl-	418007.6038	465094.8085	370920.399	331995.383	309247.809	286835.717	451991.341	351028.115	370865.797	271593.991	250433.574	261013.7825
54	Cyclohexanol, 3,3,5-trimethyl-	12015.593	14422.747	15326.731	7640.543	6824.6195	6008.696	5676.697	8876.271	7276.484	11511.548	12418.095	13324.642
55	Heptanal	8498616.58	7972833.72	7307498.77	4816380.29	6474429.61	5574717.64	6062143.24	5522424.94	5856467.36	3685587.49	3257585.35	4006036.28
56	1-Hexene, 2,4,4-triethyl-	297747.6878	264232.0025	331263.373	240398.756	244351.0105	248303.265	268081.334	215066.737	241574.0355	341202.239	288883.412	341359.611
57	Pyridinium, dinitromethylide-	393387.3485	395955.654	390819.043	399335.219	450465.229	424900.224	342311.1715	361606.236	323016.107	339960.507	286040.283	337770.626
58	1,3a-Ethano(1H)inden-4-ol, octahydro-2,2,4,7a-tetramethyl-	132598.392	147315.046	117881.738	109509.2685	114486.541	104531.996	102955.309	98125.528	88450.392	98222.767	87402.795	92812.781
59	2-Propenal, 3-phenyl-	182438.202	223560.98	202999.591	380328.622	318000.6175	255672.613	218076.7155	203447.413	232706.018	352529.704	310984.549	331757.1265
60	2-Decenal, (E)-	1171852.4	1050139.087	928425.774	1592733.03	1366567.99	1072459.28	1417483.67	1290114.14	1373364.42	1898239.29	1337140.35	1617689.82
61	2-Hexenal, (E)-	74651122.5	82171425.1	75579841.3	24667935	25859386.1	24673762.6	31914115.9	28040081.3	28631773.7	54573310.1	52549417	55202971.6
62	Carbonic acid, dimethyl ester	115884.76	108991.093	110436.149	134308.093	178172.312	150855.488	121113.005	185691.076	163647.359	325495.744	321443.279	261617.967
63	2-Propanol, 1-(2-butoxy-1-methylethoxy)-	296056.538	300615.518	292439.71	520581.538	355560.234	422992.794	513960.299	514219.266	437941.305	445453.703	516264.371	359052.777
64	2-Isopropenyl-5-methylhex-4-enal	68889.174	61429.509	53969.844	53178.381	57711.966	55445.1735	78004.132	117234.852	93775.227	181939.648	141898.492	153088.041
65	2-Undecene, 3-methyl-, (E)-	492800.965	558404.606	525602.7855	158713.311	153450.468	163976.154	203775.72	214610.788	192940.652	124151.905	112615.612	118383.7585
66	3,6-Nonadien-1-ol, (E,Z)-	574635.761	641860.526	608248.1435	281503.193	307947.148	298206.959	309417.816	278115.653	262884.112	384619.189	401518.081	479549.379

67	Succinic acid, tridec-2-yn-1-yl tetrahydrofurfuryl ester	65655.457	81777.156	73716.3065	40341.0605	48003.778	32678.343	34952.204	37301.8995	39651.595	51542.02	38812.35	45177.185
68	3-Buten-1-ol, 3-methyl-	340734.491	374088.283	359654.158	nd	nd	nd	87758.462	102272.743	103093.151	321250.656	330311.975	293960.722
69	Thiazole, 5-ethyl-2-methyl-	46167.993	51839.614	49003.8035	nd	nd	nd	nd	nd	nd	13288.2	17782.8905	15535.54525
70	1-Pentanol	4371213.11	4053713.72	3736214.33	3397906.85	3338569.75	3329499.95	2991931.96	2856785.91	2957416.73	2470562.35	2237214.07	2583767.59
71	3-Hexenoic acid, methyl ester	245674.945	210072.773	206769.652	nd	nd	nd	78006.164	66917.463	59743.139	276960.228	272151.396	264300.558
72	Glutaric acid, monoamide, N-(4-methylbenzyl)-, decyl ester	40195.844	39298.02	39746.932	14594.749	12743.275	16992.009	14184.797	12399.638	13292.2175	20636.35	20700.085	20533.367
73	1,4-Cyclohexadiene-1-methanol, 4-(1-methylethyl)-	257717.45	297865.171	277791.3105	182793.917	170758.557	163875.055	203075.85	168323.146	169178.377	157698.267	164892.688	161295.4775
74	Ylangenol	31217.774	36386.632	33802.203	25444.062	21456.882	23710.528	29312.939	25168.742	22797.884	25120.434	22845.941	29861.707
75	Acetic acid, phenylmethyl ester	581287.263	693360.431	637323.847	367678.676	367026.785	301388.617	423088.242	394437.846	410480.174	546449.038	536555.502	541502.27
76	Cedrol	451351.428	383805.0675	316258.707	295562.6415	308075.941	283049.342	318786.0335	315727.818	321844.249	405885.647	262854.683	334370.165
77	6-Methyl-6-(5-methylfuran-2-yl)heptan-2-one	4670926.06	5250547.09	4960736.575	2911609.77	3168739.7	2883801.13	3593019.16	3021472.37	3131576.11	2668742.67	2561476.23	2615109.45
78	9,19-Cyclolanostan-24-one, 3-acetoxy-25-methoxy-	13433.321	11737.901	12585.611	nd	nd	nd	5983.029	6269.5	5696.558	6563.357	8599.2235	7581.29025
79	2-(1,1-Dimethylethyl)-6-(1-methylethyl)phenol	24337.784	24338.166	24338.548	21471.5465	21950	20993.093	25104.116	23548.622	21096.218	18604.518	21096.205	19850.3615
80	Cyclohexanone, 2,2,6-trimethyl-	2685656.68	3091665.19	2428312.84	1665032.81	1888916.01	2212684.14	2166420.17	2346934.88	2215200.05	2297199.25	2091262.95	1983471
81	Dimethyl phthalate	469298.577	487759.558	478529.0675	280909.872	317167.232	244652.512	286736.08	266394.879	307077.281	235871.331	256831.778	246351.5545
82	Pyrazine, 2,6-dimethyl-	179941.354	197972.564	191127.758	174925.098	224878.145	199901.6215	1474308.59	1383358.12	1663904.52	631569.802	629907.717	763751.489
83	p-Nitrophenyl hexanoate	2412625.96	2260029.195	2107432.43	1921905.94	2325464.03	1825604.61	1588463.8	1633717.04	1951491.6	1697275.21	1607017.39	2017341.79
84	2-Penten-1-ol, (Z)-	39747358.7	37306296.4	39953931	13923868.3	14364905.5	13859087.9	13064974	12816324.7	12800893.5	38427571.4	38020170.8	36250429.9
85	2-Heptanol	118340.203	125991.068	113749.461	42393.333	36378	44370.035	32963.77	34925.339	33708.072	103643.182	101782.487	92812.513
86	4,4-Dimethyl-cyclohex-2-en-1-ol	47078.54	42975.2145	38871.889	nd	nd	nd	nd	nd	nd	95009.46	90131.917	99975.455

87	Oxalic acid, cyclohexyl dodecyl ester	71429.147	60831.797	50234.447	60331.23	81261.059	58624.303	86286.184	84187.604	95794.149	85279.269	119282.5565	102280.9128
88	2-Hexen-1-ol, acetate, (Z)-	100169.123	99684.584	99200.045	nd	nd	nd	nd	nd	nd	85429.754	74351.5	96508.008
89	1,7-Octadiene-3,6-diol, 2,6-dimethyl-	514299.404	605389.931	559844.6675	235862.748	273960.877	254911.8125	331747.43	403345.381	367546.4055	599113.413	508604.564	553858.9885
90	1-Tetradecene	120081.98	114077.31	117079.645	34988.914	40640.586	44021.848	57165.4705	63841.685	50489.256	63094.543	40444.554	51769.5485
91	Propanoic acid, 2-methyl-, hexyl ester	132445.681	125882.934	119320.187	49230.689	65368.537	57299.613	50029.985	52501.888	54973.791	77963.113	98710.355	119457.597
92	5-Heptenal, 2,6-dimethyl-	825744.214	787282.8585	748821.503	604996.937	712679.095	604038.52	483356.589	514544.644	549554.827	415758.576	411344.074	546277.296
93	Cyclopentanone, 2,4,4-trimethyl-	85058.734	95754.23	80996.703	61879.131	75545.996	68712.5635	43235.678	49496.895	44851.539	38409.173	32964.124	35686.6485
94	(+)-Borneol	249320.341	229692.774	255937.258	94269.625	117132.143	98583.881	99890.476	111738.24	105379.489	186351.519	186092.709	188284.926
95	2-Methyl-2,5-divinyltetrahydrofuran	111729.557	111756.665	111702.449	79565.308	98424.79	78125.785	nd	nd	nd	nd	nd	nd
96	1-Hexanol	27289582.8	25543907.3	27075422.1	10218110	10612542.5	10930397.1	8558527.7	8724592.13	8696225.75	22570134.4	22151467.3	20111300
97	1H-Inden-1-one, octahydro-7a-hydroxy-	272981.545	228943.4525	250962.4988	174645.326	170466.654	172584.63	285125.233	253202.939	246528.373	201625.186	171370.638	186497.912
98	3-Nonanol	129486.601	125390.273	114437.298	nd	nd	nd	26907.086	24365.05	25636.068	49631.947	61234.865	55433.406
99	6-Methylhept-4-en-1-yl 2-methylbutanoate	198399.695	175655.3515	152911.008	222831.8295	219816.271	225847.388	210106.041	264612.266	226026.325	223205.819	171272.529	197239.174
100	4-Pyridinamine, N,N-dimethyl-	179482.228	168623.414	140249.705	68564.305	85040.273	66751.166	647214.466	582405.629	697459.782	644108.976	665441.487	822714.221
101	3-Hexen-1-ol, (E)-	68912378.9	64793124.7	69242813.9	25595679.6	28272581.7	27747744.1	24915076.1	25384907.7	25233648.2	60359725.7	60182352	56851350.9
102	Tetrahydrofuran, 2-isobutenyl-4-vinyl-	258168.911	299989.295	303766.927	259860.765	296912.771	222808.759	228049.7115	220939.643	235159.78	185527.041	146636.574	166081.8075
103	Benzeneethanol, 2-hydroxy-	68755.471	88281.752	78518.6115	65589.334	45420.064	52280.267	63359.128	53296.499	60870.991	38043.122	44701.019	41372.0705
104	Hexanoic acid, hexyl ester	146411.843	140135.057	132967.233	94751.7945	112460.196	77043.393	92250.642	100836.85	109423.058	95850.306	113760.135	104805.2205
105	2-Propenal, 2-methyl-3-phenyl-	26298.726	33138.188	29718.457	nd	nd	nd	nd	nd	nd	nd	nd	nd
106	2-Hexen-1-ol, (E)-	72770244.2	68460373.8	71090182.8	25846023.4	28201937.2	27845131.7	22259287.7	22666700.7	22381984.2	62468298.8	61990588	58820581.3
107	5-Ethylcyclopent-1-enecarboxaldehyde	266664.469	299227.819	240263.922	230031.027	256704.764	260785.931	211413.113	199121.3	203257.309	267520.744	256968.67	268434.993
108	2-Cyclopenten-1-one, 2,3,4,5-tetramethyl-	90522.982	84108.8835	77694.785	61104.205	65808.857	55751.705	71108.479	72205.905	70011.053	58427.969	48034.79	53231.3795
109	2-Hexen-1-ol, (Z)-	1102787.13	1028578.28	1086968.25	341901.352	394871.807	391639.975	320642.198	323552.129	324727.04	902373.057	887538.584	840488.765
110	Fumaric acid, heptyl myrtenyl ester	12652.504	17036	14844.252	nd	nd	nd	nd	nd	nd	nd	nd	nd

111	Decanal	252223.3403	300023.6295	276123.4849	nd	nd	nd	nd	nd	nd	nd	nd	nd
112	3-Hexanol, 2-methyl-	142839.549	139205.237	135634.25	145261.394	159324.406	135449.912	185389.632	188051.545	189766.798	123953.565	126120.18	120952.878
113	Methional	510518.255	485247.124	509878.987	438822.581	485370.711	416152.085	434221.433	405866.383	444255.531	441444.409	456318.813	477650.751
114	Eicosyl heptyl ether	24438.069	19799.468	22118.7685	nd	nd	nd	8596.3025	10161.749	7030.856	nd	nd	nd
115	1-Octen-3-ol	13709588.9	14222229.6	13965909.25	6478635.03	8564678.87	7548297.8	8354490.4	8419380.16	8458318.43	9744278.85	8154127.61	8949203.23
116	Furfural	4787471.86	4896533.9	4391231.33	2706219.78	3130895.1	2460702.61	5091256.38	4931300.45	5406548.96	15367825.5	16424308.6	18758600.5
117	2-Octanone	9236822.58	8519428.45	9954216.71	17196673.4	13186997.7	19244473.9	16147302.8	17951373.5	15600421.1	18112254	14996314.7	16554284.35
118	3-Methyl-hexahydrophthalide	2889188.54	2567335.795	2245483.05	828208.228	957228.87	1067368.59	1026842.21	1015349.43	957338.187	1596035.67	1447838.58	1521937.125
119	(R,S)-5-Ethyl-6-methyl-3E-hepten-2-one	432529.646	445294.446	438912.046	218852.158	221644.886	251824.539	243548.983	212640.192	228094.5875	256990.567	249500.955	253245.761
120	6-Hepten-1-ol, 2-methyl-	1940239.23	1928618.69	1732380.09	570036.983	633691.558	650096.649	550526.419	516750.07	516227.662	1415336.82	1379370.01	1402881.5
121	2H-Pyran, 3,6-dihydro-4-methyl-2-(2-methyl-1-propenyl)-	49722.869	46241.243	42759.617	nd	nd	nd	39344.052	39937.526	40531	33835.359	28312.989	31074.174
122	1H-Pyrrole, 1-ethyl-	269700.298	183692.158	226696.228	138222.661	145318.141	124770.75	302017.852	243337.452	256041.243	605918.695	599738.703	603733.549
123	p-Mentha-1(7),8(10)-dien-9-ol	326805.01	386426.493	356615.7515	203781.528	191310.057	197925.943	190121.845	175234.454	175726.844	216986.596	212305.617	265494.368
124	Ethyl 2-(5-methyl-5-vinyltetrahydrofuran-2-yl)propan-2-yl carbonate	96754323.4	93626074.5	92711336.7	50864707.9	57492585.2	52092255.8	50509823.7	49966569	50809918	76425112.3	77496848.7	79824528.3
125	2-Butenal, 2-ethyl-	697062.696	1021876.95	859469.823	239215.895	298029.374	317070.689	657010.458	542356.514	534788.817	2638758.36	2433405.94	2619938.77
126	1,6,10-Dodecatrien-3-ol, 3,7,11-trimethyl-, (E)-	946525.576	845614.058	744702.54	588748.147	597310.176	577763.488	548478.677	541436.5	555520.854	436491.823	441470.709	438981.266
127	Octanal	1325856.52	1117241.612	908626.704	501007.208	651735.939	659284.091	765599.525	662350.787	689604.233	702432.386	674610.956	688521.671
128	4-Heptenal, (Z)-	318146.587	268300.468	218454.349	163438.717	168207.412	159286.087	205512.591	167936.27	163141.684	201767.723	182006.756	172003.313
129	Benzaldehyde, 2,5-dimethyl-	667809.329	646019.226	624229.123	482167.682	540851.278	556328.892	746342.803	761371.497	721514.297	828002.628	812292.938	970896.181
130	2-Furanecarboxylic acid, 3,5-dimethylcyclohexyl ester	742989.89	823592.975	625103.077	466985.793	535783.187	455667.324	918687.504	892083.392	985888.937	1371425.4	1453510.55	1609538.81
131	1H-Pyrrole-2-carboxaldehyde	340224.682	318217.4705	296210.259	157400.543	149218.883	122021.547	423813.915	371915.163	414019.92	1385955.45	1477415.57	1431685.51

132	3-Cyclopentylpropionic acid, 1-(cyclopentyl)ethyl ester	106688.019	101998.3	98984.597	94580.005	93246.399	92614.607	111268.483	114059.59	117674.809	125649.54	138332.34	131175.803
133	3-Ethyl-4-methylpentan-1-ol	165144.886	185023.594	175084.24	74388.652	97109.744	97725.276	88219.759	79713.644	88103.351	130620.908	106715.045	120318.82
134	Benzaldehyde	72968940.3	74068442.4	61982295.9	42577355.3	49529377.1	41276321.7	44350600.9	41839718	45087723	51933440.1	52774537.6	64487905.6
135	Benzoic acid, 2-formyl-4,6-dimethoxy-, 8,8-dimethoxyoct-2-yl ester	1185458.9	966591.126	1045365.07	1078662.99	1044780.78	1140405.96	1048919.2	1052063.76	922926.476	902813.646	818237.476	860525.561
136	3,5-Octadien-2-one, (E,E)-	3172092.38	3437512.27	2568273.92	1572548.62	1900907.39	1768131.48	1638892.53	1580315.5	1614091.49	2116889.55	2060299.65	2417674.76
137	5-Hexenal, 4-methylene-	186393.825	210543.852	177165.094	86059.855	96420.205	86415.446	84943.059	76962.306	90644.866	137838.522	139959.025	181832.425
138	2-Pentene-1,4-dione, 1-(1,2,2-trimethylcyclopentyl)	651976.759	730459.908	569080.9	386395.853	495164.677	459077.856	495422.737	474688.673	474709.442	465162.994	436389.495	532383.83
139	2-Dodecenal	499882.698	450611.425	401340.152	266401.736	346389.964	298925.393	348829.6	329812.603	303859.094	308721.779	294059.254	301390.5165
140	cis-Muurola-4(15),5-diene	402273.677	412194.502	422115.327	nd	nd	nd	389736.602	358760.413	317153.715	261197.388	212217.527	236707.4575
141	Octadecanoic acid, ethenyl ester	354094.735	282456.439	303758.344	338788.633	318658.03	331892.324	320232.074	275570.484	293778.928	261275.534	251480.951	252577.755
142	Linalool	104796021	114343387	89670706.9	44111638.4	50332067.3	55110911	51527312.5	46767825.7	45957367	73323868	71412917.1	78894298.3
143	Orcinol	857497.036	980570.606	729660.011	628696.633	690719.876	631517.822	839876.414	772587.152	819136.792	557391.395	542761.956	614133.026
144	2-Cyclohexen-1-ol, 2,4,4-trimethyl-	91628.182	98380.842	86034.75	74087.462	86210.421	83758.662	93118.415	88821.593	88770.916	87876.613	82252.73	81072.339
145	(+)-2-Bornanone	319708.711	296126.9265	272545.142	364497.33	321989.499	279481.668	238055.4105	215360.392	260750.429	269318.237	259304.358	264311.2975
146	Ethanone, 1-(2-methyl-1-cyclopenten-1-yl)-	477562.822	507234.746	432671.318	301314.519	329273.406	263696.31	621192.605	615034.966	672680.827	1548524.72	1731979.06	2015901.2
147	2,6-Octadiene-1,8-diol, 2,6-dimethyl-	111673.683	115019.393	115603.078	116528.027	145418.7	130973.3635	107375.816	131012.144	154648.472	270816.217	236015.599	218604.923
148	2-Heptenal, (Z)-	4091135.52	3764139.825	3437144.13	3462737.07	5047120.25	4467602.14	5696489.115	5706428.98	5686549.25	6855891.29	5558659.55	6207275.42
149	1-Cyclohexene-1-carboxaldehyde, 2,6,6-trimethyl-	4395318.98	5023391.51	4709355.245	2155333.45	2898226.2	2711609.28	2886717	2742923.63	2791000.47	2496927.37	2308260.03	3138492.5
150	1H-Pyrrole-2,5-dione, 1-ethyl-	148856.754	138628.1305	128399.507	227993.2305	239640.252	216346.209	233122.83	245552.948	250819.32	372630.021	414382.454	393506.2375
151	2,6-Nonadienal, (E,Z)-	723870.117	633536.15	543202.183	328576.375	391984.628	356780.549	439612.408	373434.952	384513.982	397498.229	411666.468	404582.3485
152	Lilac aldehyde D	54942.74	57210.005	49836.403	nd	nd	nd	31142.125	31331.787	31521.449	nd	nd	nd

153	2-(3-methyl-2-cyclopenten-1-yl)-2-methylpropionaldehyde	nd	nd	nd	nd	nd	nd	58164.145	63294.383	61914.86	93570.904	87456.002	90513.453
154	Cyclopropanecarboxylic acid, 2-methyl-, 2,6-di-t-butyl-4-methylphenyl ester	928236.758	857183.37	696775.811	384410.641	369027.02	328578.14	314066.496	341413.056	310446.301	394316.287	405896.495	430612.623
155	2-Octenal, (E)-	205346.0555	226033.126	184658.985	nd	nd	nd	nd	nd	nd	nd	nd	nd
156	3-Buten-2-one, 4-(2-hydroxy-2,6,6-trimethylcyclohexyl)-	170836.248	176475.378	173655.813	74019.9995	72626.605	75413.394	74221.439	60178.105	67199.772	nd	nd	nd
157	2,6-Dimethyl-2-trans-6-octadiene	1331272.07	1185384.355	1039496.64	926524.495	1137196.513	1347868.53	1728715.99	1718428.83	1740105.87	1838315	1373213.85	1605764.425
158	Acetylacetone	25360.685	34175.326	29768.0055	17611.121	12141.304	14876.2125	22604.497	19250.886	15897.275	nd	nd	nd
159	Ethanone, 1-(1,4-dimethyl-3-cyclohexen-1-yl)-	200971.414	189869.246	168853.047	105180.718	140844.586	118373.86	129405.905	105359.35	106688.196	81933.958	82687.919	82310.9385
160	1H-Pyrrole-2-carboxaldehyde, 1-ethyl-	3841440.35	4131340.44	3529791.36	1492123.11	1677880.59	1419825.38	5261004.47	5140371.32	5473944.81	14881478.3	15834944.9	17146703.5
161	1H-Cycloprop[e]azulene, decahydro-1,1,7-trimethyl-4-methylene-	38209.641	39190.647	40171.653	42089.189	38626.139	37825.897	50857.63	46887.102	48726.021	37450.223	36707.274	37078.7485
162	1,5,7-Octatrien-3-ol, 3,7-dimethyl-	15469026	17046683.8	13035122.1	9287429.52	10421148.4	8841426.8	12771131.4	11421462.1	11786228.2	19443832.4	19458102.2	22747456.6
163	Acetaldehyde	767982.071	687644.653	740322.187	451065.66	641056.433	621829.041	676223.707	665355.214	625049.899	680700.094	768604.849	738797.073
164	Fumaric acid, cyclohex-3-enylmethyl isohexyl ester	77795.912	88061.399	65264.895	32318.957	32958.798	34977.562	35025.328	35445.7855	35866.243	65078.803	66728.24	74165.666
165	Resorcinol	197078.792	136621.263	166850.0275	88672.638	85000.512	81880.826	64558.688	67325.153	61792.223	57817.407	59444.379	60308.773
166	2,4-Heptadienal, (E,E)-	17374740.3	15395784.6	16385262.45	7239981.63	7998147.57	6970010.75	6676182.38	6233916.11	6433949.59	8543350.65	8477345.13	10586204.3
167	Ethanol, 2-(2-ethoxyethoxy)-	161314.195	166587.596	163950.8955	128392.423	118699.454	98729	121583.236	113360.714	94589.127	118824.024	109213.852	114018.938
168	Bicyclo[4.4.0]dec-1-ene, 2-isopropyl-5-methyl-9-methylene-	288646.488	254339.9795	220033.471	197726.419	171937.347	172695.891	175400.868	155045.516	145879.743	141979.012	131473.632	136726.322
169	Isobutyl laurate	22760.408	26287.392	20167.159	18321.773	15053.482	12751.303	nd	nd	nd	13260.97	15807.1965	18353.423
170	Benzenamine, 4-methoxy-2-methyl-	145520.652	156523.938	126989.377	nd	nd	nd	257621.603	237493.594	257273.225	362391.622	356450.857	455562.889

171	2,6-Dimethyl-8-(tetrahydropyran-2-yloxy)-octa-2,6-dien-1-ol	nd	nd	nd	nd	nd	nd	33679.137	31302.9755	28926.814	31677.906	26827.224	29252.565
172	1,3-Cyclohexadiene-1-carboxaldehyde, 2,6,6-trimethyl-	499430.676	441615.9265	383801.177	277883.353	329312.443	341614.533	508397.853	491192.438	473219.816	570445.862	545733.58	671161.111
173	Furan, 3-methyl-	242993.86	265528.322	202172.85	140428.235	187596.022	200802.145	210276.476	203103.323	184423.771	219210.992	160451.096	181579.339
174	2-(Prop-2-enoyloxy)tridecane	118265.596	148001.724	142898.518	75895.649	65166.012	62427.136	90832.251	74144.249	76370.295	86783.244	81676.739	103166.188
175	Pentanoic acid, 2-methyl-, anhydride	538162.888	610961.613	574562.2505	250012.001	292177.1395	334342.278	298772.774	282690.967	225140.663	383348.55	377287.161	380317.8555
176	Hexanoic acid, 3-hexenyl ester, (Z)-	4269210.69	3871814.24	3474417.79	1922491.53	2015310.82	1931769.72	2452096.4	2124157.64	2064012.15	2268227.88	1980352.84	2124290.36
177	Ethanone, 2-(3-nitro-[1,2,4]triazol-1-yl)-1-thiophen-2-yl-	96896.896	89719.8805	82542.865	58145.272	71377.487	64761.3795	nd	nd	nd	84046.816	85256.505	96896.034
178	3-Hexenal	932150.893	948186.412	916115.374	447399.993	418354.412	426932.402	453195.01	444834.36	461555.66	600571.516	593585.13	646805.328
179	Hydratropic acid, butyl ester	90416.23	79631.915	68847.6	92676.367	96867.69	88485.044	62192.349	57930.915	66453.783	69122.866	70595.848	69859.357
180	1-Nonanol	1310414.15	1627757.72	1469085.935	576402.902	619308.073	645965.415	732245.572	628944.547	630510.682	738769.038	681565.562	710167.3
181	1-Octanol	2384936.5	2025038.765	2204987.633	1333620.86	1541695.7	1487506.5	1614643.8	1431524.19	1469538.45	1558929.2	1415187.4	2027409.22
182	trans-2-Hexenyl isovalerate	1082697.66	931405.9965	780114.333	277611.248	360315.519	398066.796	520250.315	501261.664	448122.984	471254.917	394423.963	432839.44
183	Hexanoic acid, 2-hexenyl ester, (E)-	1786240.55	1613443.495	1440646.44	847624.724	900325.672	844521.507	1109539.44	963578.73	938832.486	1014490.13	869164.75	941827.44
184	Butanoic acid, 3-methyl-	3804649.07	3756772.24	3659718.68	2782697.47	2623983.955	2465270.44	2804336.75	2633876.73	2851063.48	2477254.03	2559428.13	2991073.14
185	2,6-Octadienal, 3,7-dimethyl-, (Z)-	1323780	1196245.625	1068711.25	663524.442	661370.998	654714.808	685478.382	576822.307	600937.048	761550.388	749150.854	755350.621
186	Formic acid, phenylmethyl ester	195946.9	251466.451	196208.313	165755.645	142724.802	133180.961	166410.596	151468.572	162634.432	224771.333	225159.6	301455.573
187	Bicyclo[3.1.1]hept-2-en-6-one, 2,7,7-trimethyl-	55171.519	52811.017	50450.515	36302.194	34020.044	34221.302	40929.584	38204.3615	35479.139	29489.143	40695.377	35092.26
188	3-Thiophenecarboxaldehyde	146565.118	158439.618	126531.598	84767.506	100064.931	86481.522	144317.008	137570.914	140362.4	160390.271	175930.202	201950.659
189	2-Octen-4-one, 2-methoxy-	150135.077	214789.268	182462.1725	nd	nd	nd	nd	nd	nd	nd	nd	nd
190	Carotol	463455.791	416411.7355	369367.68	395112.003	392083.19	393597.5965	347705.99	281427.332	316574.526	346964.447	297034.829	321999.638
191	2-(1-Pyrrolidinyl)ethyl 4-propoxysalicylate	478100.604	565443.588	521772.096	nd	nd	nd	nd	nd	nd	nd	nd	nd

192	2(3H)-Furanone, 5-ethyldihydro-	2852863.32	3011681.34	2499051.16	1913452.07	2169518.76	1771627.43	1921218.52	1829156.27	1920392.96	2153361.39	2212576.06	2562976.13
193	trans-Geranic acid methyl ester	425303.446	378292.608	331281.77	155551.166	178377.164	193785.67	231215.117	213398.367	201796.117	224891.61	208902.456	216897.033
194	1-Methylverbenol	464569.121	488622.169	403350.201	233660.152	269858.828	250903.898	251500.977	223339.288	226886.376	302015.134	313093.943	331230.285
195	7-Octen-2-ol, 2-methyl-6-methylene-	85370.362	79374.1205	73377.879	79080.729	64234.888	63326.983	65450.571	51923.033	58686.802	70895.039	71801.788	71348.4135
196	2-Methyl-7-oxabicyclo[2.2.1]heptane	750378.856	524932.473	637655.6645	335030.9	375469.573	375941.939	578993.771	535570.412	557118.292	563558.973	433815.418	498687.1955
197	2-Hexene, 3,5,5-trimethyl-	3863365.87	3285196.015	2707026.16	2087703.62	2801133.65	2395400.77	2842363.15	2909211.89	2951157.21	3251294.47	2642345.1	2946819.785
198	(+)-epi-Bicyclosesquiphellandrene	649575.227	592816.48	536057.733	486402.18	409345.108	364749.191	307226.794	316290.003	297458.876	247545.54	268499.89	311614.999
199	2,4,8-Trimethyl-1,2,3,4-tetrahydroquinoline	116701.339	109464.385	113082.862	75151.171	57726.204	54453.602	44592.8405	48626.284	40559.397	41202.961	43127.6945	45052.428
200	2-Cyclohexene-1-methanol, 2,6,6-trimethyl-	320958.588	321291.17	282374.224	208149.044	241528.957	185401.815	187568.952	196012.948	196766.779	192995.26	217390.105	279381.87
201	Hexanoic acid, methyl ester	965098.544	839636.711	758507.787	235108.2745	209705.316	260511.233	339643.931	371479.539	307808.323	900476.175	785554.316	771565.224
202	2-Cyclohexen-1-one, 3-methyl-6-(1-methylethyl)-	281965.297	265428.4125	248891.528	681516.604	553387.486	617452.045	434221.089	360177.567	422783.735	941039.265	537677.206	739358.2355
203	3,4-Dimethyl-3-pyrrolin-2-one	4858961.97	4353567.525	3848173.08	7288687.87	5127523.72	6782497.21	6667619.83	7915097.88	7524804.31	9376233.76	11050556.7	10213395.23
204	2,6-Octadienal, 3,7-dimethyl-, (E)-	11753260.5	10492483.14	9231705.78	6518501.56	6304174.22	6160779.4	6532461.17	5555761.1	5673790.06	6489035.98	6405826.98	8629214.13
205	Hexanoic acid, ethyl ester	185231.77	163343.366	141454.962	96934.535	124692.83	112306.983	89073.2065	91556.389	86590.024	82158.957	67635.556	88986.545
206	Phenol, 4-propyl-	251675.258	309962.504	220010.873	242382.333	221306.703	200231.073	194736.957	194245.576	181404.109	130049.312	152977.123	141513.2175
207	Fumaric acid, isobutyl undec-2-en-1-yl ester	215125.103	225341.162	188786.266	122103.178	146393.973	115369.578	131371.253	127526.528	123681.803	149145.2595	140371.141	157919.378
208	4-Amino-2,3-xylenol	94428.442	110282.923	86348.026	31862.328	29069.816	24960.817	158590.389	157195.133	173817.478	380750.511	406163.958	517147.418
209	(E)-4-Oxohex-2-enal	3088654.28	3363420.84	2658164.44	2021129.28	2307674.99	1934208.32	2215181.34	2068959.18	2216764.39	1866381.04	1885531.71	2357022.35
210	2-Undecenal	248069.497	221918.0285	195766.56	164521.614	178408.882	150187.009	220227.424	203794.974	215039.104	257952.65	217786.648	237869.649
211	Benzamide, N-[4-[7-(benzoyloxy)-2-oxo-2H-1-benzopyran-3-yl]phenyl]-	851474.258	769581.0045	687687.751	737908.685	617813.225	517691.179	746114.767	598062.86	640922.871	622236.882	629075.37	625656.126
212	Benzeneacetaldehyde	235274422	219115250.5	202956079	170407231	182256235	157891694	188364350	167332494	186457692	170159856	180139525	231361694

213	Senkyunolide H	26334.699	26202.895	26071.091	nd	nd	nd	nd	nd	nd	nd	nd	nd
214	Cyclopenta[c]pentalen-3(3aH)-one, octahydro-1,2,3a,6-tetramethyl-	59670.886	63957.514	61814.2	55379.32	47121.209	38863.098	40117.3275	41202.008	39032.647	41424.08	37988.608	39706.344
215	(d-Threo-2,3-diphenyl)-2-butanol	948901.516	1065732.59	845064.385	784940.115	774733.676	635237.926	928939.769	741404.959	710561.511	814232.147	778594.193	1054078.82
216	2-Propylphenol, n-propyl ether	110573.051	141168.452	125870.7515	48367.4585	45828.022	50906.895	60957.875	53804.563	54205.228	52016.775	49592.995	50804.885
217	2-Pyrazoline, 5-ethyl-1,4-dimethyl-	72770.827	72914.436	72842.6315	34614.216	32198.3985	29782.581	43600.925	36504.793	39341.938	40904.022	41966.646	41435.334
218	(3R,6S)-2,2,6-Trimethyl-6-vinyltetrahydro-2H-pyran-3-ol	52787070.9	54572081.9	46579709.1	32047446.4	38075574.7	31344991.1	34185565.4	31924553.9	33776013.1	34637605.4	35638921.3	41484810
219	Methyl salicylate	59049929.7	71913678.1	57573260.3	35927738.7	34515219.5	35503988.5	32500988.6	30617992.2	30178248.6	53510680.1	55902426.7	64802388.4
220	Citronellyl butyrate	313692.004	233521.023	273606.5135	nd	nd	nd	nd	nd	nd	nd	nd	nd
221	3-Hexen-1-ol benzoate	113819.22	105971.6655	98124.111	110408.818	82402.431	82868.701	102351.101	86126.367	92096.022	89144.191	78743.424	83943.8075
222	2-Hexenoic acid, 3-hexenyl ester, (E,Z)-	119067.74	112341.3755	105615.011	59338.687	64318.891	48173.371	67950.8195	67030.237	68871.402	69191.366	65642.318	67416.842
223	6-Octen-1-ol, 7-methyl-3-methylene-	4189447.57	4317017.05	4253232.31	1682811.25	1819086.37	1885163.15	1989124.51	1686393.64	1690770.05	2027808.58	2033692.44	2513302.33
224	6-Propenylbicyclo[3.1.0]hexan-2-one	362437.745	405561.077	383999.411	153680.888	172471.377	175387.132	184627.469	166318.901	161148.886	184760.589	187153.546	242054.128
225	1H-Pyrrole-3-carbonitrile	256220.988	280982.697	268601.8425	nd	nd	nd	nd	nd	nd	317327.265	242780.9435	280054.1043
226	2,5-Cyclohexadien-1-one, 2,6-bis(1,1-dimethylethyl)-4-hydroxy-4-methyl-	85088.04	72548.9905	60009.941	108650.4425	105456.249	111844.636	117358.859	94523.41	94420.192	91459.685	92657.896	92058.7905
227	Pyrazine, ethyl-	345427.915	334188.346	344006.567	100708.748	137858.233	107681.178	634824.235	605720.153	724409.762	2175514.04	2291669.78	2706298.82
228	Cyclohexanol, 1-methyl-4-(1-methylethylidene)-, acetate	92926.459	81939.2755	70952.092	nd	nd	nd	nd	nd	nd	nd	nd	nd
229	2,4-Decadienal, (E,E)-	361223.13	442048.778	401635.954	236103.498	226876.49	219388.32	256255.29	223017.801	221786.765	249499.047	240125.409	244812.228
230	Ethyldiethanolamine, O,O'-diacetyl	797496.798	680818.5345	564140.271	437401.28	425569.841	432964.664	468876.46	449052.094	438686.047	413116.506	365697.075	431930.105
231	Diethyl Phthalate	111667.269	128241.145	119954.207	85501.0465	88526.31	82475.783	157435.733	129363.888	149841.512	130004.432	131208.538	130606.485
232	2-Buten-1-one, 1-(2,6,6-trimethyl-1-cyclohexen-1-yl)-	139466.091	111412.188	107082.236	100936.097	109209.77	88486.377	80125.411	76853.444	77656.775	53353.415	50427.108	51890.2615
233	Anethole	1721347.75	1402818.33	1562083.04	3180770.49	2963118.18	2660485.83	2411277.03	2005186.28	2222522.01	3246639.65	2826574.47	3036607.06

234	1H-Benzimidazole, 2-methyl-	23682.448	20939.1485	18195.849	nd	nd	nd	15556.052	16373.356	14738.748	nd	nd	nd
235	Lilac alcohol B	175931.115	155268.851	134606.587	91777.565	107993.614	91353.659	107035.319	96034.017	99868.647	96319.014	100596.655	98457.8345
236	1,3-di-iso-propylnaphthalene	186464.611	194582.842	190523.7265	129897.478	106942.995	93021.809	65913.3405	69625.945	62200.736	63448.941	61016.982	67619.762
237	(E)-2,6-Dimethylocta-3,7-diene-2,6-diol	11699285.7	10228233.2	8757180.69	7577174.86	7601985.48	6401860.09	7232745.965	6853516.7	7611975.23	5525561.26	5400969.56	5463265.41
238	2,6,6-Trimethylcyclohexa-1,4-dienecarbaldehyde	224252.614	289380.949	256816.7815	150615.591	162257.476	154736.307	154594.363	134388.77	140931.563	148146.616	151225.762	149686.189
239	2-Butanone, 4-(4-methoxyphenyl)-	43228.486	46530.578	51412.287	29784.109	29166.9775	28549.846	27060.626	27503.9815	27947.337	nd	nd	nd
240	Isophorone	545003.29	668392.674	606697.982	352107.344	487185.566	470630.019	519786.767	548987.937	549734.393	551279.034	442231.303	480457.507
241	7-Isoquinolinol, 1,2,3,4-tetrahydro-1-[(3-hydroxy-4-methoxyphenyl)methyl]-6-methoxy-2-methyl-, (S)-	nd	nd	nd	nd	nd	nd	nd	nd	nd	107439.139	94833.718	101136.4285
242	Butanal, 2-methyl-	37906157.7	37009192.6	37457675.15	40754634	33602503.4	44017230.8	52997732.2	53821536.8	53337310.8	58854447.9	52654274.8	53028944.1
243	3-(Hydroxyimino)-6-methylindolin-2-one	64411.686	65590.295	65000.9905	nd	nd	nd	nd	nd	nd	42192.013	41521.391	41856.702
244	3-Methyl-2-(3-methylpentyl)-3-buten-1-ol	470695.732	423637.12	376578.508	283165.226	338749.528	265738.935	382774.445	319607.325	324144.527	228378.931	218703.968	223541.4495
245	(Z)-Hex-3-enyl isobutyl carbonate	173775.14	130331.355	152053.2475	121172.884	115775.669	119743.514	nd	nd	nd	134698.835	117517.981	126108.408
246	2H-1-benzopyran-6-ol, 3,4-dihydro-2,2-dimethyl-4-(1-methylethyl)-	216520.654	217322.789	224939.847	199010.958	214653.407	206832.1825	120979.713	104602.207	110575.736	107707.819	97447.668	102577.7435
247	1H-Indole, 2,3-dihydro-4-methyl-	36568.966	44328.845	40436.891	46797.871	33613.188	38687.012	35193.938	32530.138	35002.164	25928.124	28023.168	26975.646
248	Benzyl alcohol	87261069.3	93235536.3	73393129.2	58736178.1	63873248.5	55594493.8	64238107.5	60194494	62541555.7	58762446.1	60890926.5	73027411
249	2,5-Pyrrolidinedione, 1-ethyl-	2896215.18	3377225.81	3136720.495	2013335.99	1818619.6	1835132.1	3092534.75	2491376.71	2631844.49	1990269.21	1990384.42	1990326.815
250	Octanal, 7-hydroxy-3,7-dimethyl-	66000.796	56359.758	46718.72	nd	nd	nd	nd	nd	nd	nd	nd	nd
251	5-Aziridinopentanol	77962.411	69705.703	73834.057	54932.716	67868.465	61400.5905	55241.443	47814.932	51528.1875	46701.14	50618.865	48660.0025
252	2,6-Octadienoic acid, 3,7-dimethyl-, (E)-	30419390.8	38478165.6	34448778.2	13680416.5	12062556.5	16669724.3	20040588	22390457.4	17690718.6	13674879	12205016.2	12939947.6

253	Fumaric acid, isohexyl pent-4-en-2-yl ester	100408.209	88853.369	90316.734	67881.167	61556.64	55232.113	68168.07	67732.976	67297.882	57935.325	61223.931	59579.628
254	3,7,11-Trimethyl-dodeca-2,6,10-trienoic acid	489440.24	539653.988	514547.114	226203.914	248967.672	250702.76	299676.927	247786.715	258444.218	238470.644	220792.169	229631.4065
255	(3R,3aR,3bR,4S,7R,7aR)-4-Isopropyl-3,7-dimethyloctahydro-1H-cyclopenta[1,3]cyclopropa[1,2]benzen-3-ol	173547.957	153085.2355	132622.514	94387.433	109943.809	124844.72	127954.4	121411.85	104642.254	77597.69	64972.179	71284.9345
256	Phenylethyl Alcohol	91462554.7	101647723	78774102	66130735.8	70565730.3	62526101.3	71266795.8	64985965.1	68000514.5	64102987.1	65820802.5	81704918.6
257	Propanoic acid, 2-methyl-, 2-methylbutyl ester	844003.465	940600.686	724364.543	297830.724	248605.771	347055.677	555038.113	502473.3525	449908.592	322090.495	235654.18	320142.376
258	(1R)-2,6,6-Trimethylbicyclo[3.1.1]hept-2-ene	5778663.51	5540104.59	5301545.67	3076728.975	3003148.51	3150309.44	3549764.86	3106086.78	2785587.57	1996565.87	1842285.285	1688004.7
259	7-epi-a-Eudesmol	542723.076	492747.5125	442771.949	387973.707	355512.743	370448.148	409662.936	355818.662	338804.033	293988.389	269111.307	281549.848
260	Terephthalic acid, hexyl tridec-2-yn-1-yl ester	105134	108780	107106	109611.4315	111380.001	107842.862	107415	106895	106207.117	105588.999	104146	102904.649
261	Neophytadiene	114475.0005	114036.531	114913.47	156270.259	130939.626	128994.143	162320.853	140535.972	146258.278	143826.916	127701.605	135764.2605
262	2,9-Heptadecadiene-4,6-diyn-8-ol, (Z,E)-	219365.417	193912.111	168458.805	87295.852	95908.633	104419.821	187615.693	165124.213	166974.833	203791.464	202509.749	203150.6065
263	Cyclopentanecarboxylic acid, 2-tetrahydrofurylmethyl ester	76248.573	65250.085	54251.597	nd	nd	nd	72000.39	74668.682	70027.812	100244.624	75091.62	87668.122
264	2-Cyclopenten-1-one, 3-methyl-2-(2-pentenyl)-, (Z)-	3134785.37	2820322.07	2505858.77	2199141.11	2231056.21	2160728.93	2635640.09	2232743.78	2313483.66	2208793.33	2238380.41	2223586.87
265	Homovanillyl alcohol	85222.251	73599.522	79410.8865	44280.772	38013.0385	31745.305	nd	nd	nd	nd	nd	nd
266	Tricyclo[6.3.0.0(2,7)]undecane-1,3,7-triol	27011.195	23972.9585	20934.722	17534.916	19942.172	15127.66	33128.569	30497.981	31757.1	28415.896	22424.372	25420.134
267	Hexanoic acid, 2-ethyl-	1128571.82	1246233.22	956031.049	1167147.12	1131394.13	1226617.66	1389137.91	1274557.65	1228369.92	1278307.46	1264209.05	1301932.71
268	Ethyl (E)-hex-3-enyl carbonate	1105989.85	1388757.17	1247373.51	773394.859	773722.022	689846.947	nd	nd	nd	727176.746	719177.737	723177.2415

269	1-Hepten-3-one	216145.273	211165.708	213655.4905	61505.965	61558.114	74204.035	64855.386	60845.405	68865.367	79774.628	72919.646	76347.137
270	2,7-Octadiene-1,6-diol, 2,6-dimethyl-	237038.275	213567.946	190097.617	190258.225	141343.833	148978.593	207548.23	157051.218	171270.862	123635.132	129467.856	126551.494
271	Ethanone, 1-(1H-pyrrol-2-yl)-	658253.246	813665.666	735959.456	nd	nd	nd	nd	nd	nd	1999740.85	2230081.31	2114911.08
272	2-Hexenoic acid, (E)-	49060574.1	43707600.75	38354627.4	24281538.3	27334520.9	23588515.9	33449199	28814103.4	31652574.2	23915711.9	24541710.5	24228711.2
273	Hexenyl tiglate, 4Z-	64518.794	73370.179	58688.799	40189.321	41649.273	42862.581	nd	nd	nd	nd	nd	nd
274	Naphthalene, 1,6-dimethyl-4-(1-methylethyl)-	1046198.99	1098666.55	837307.877	827154.878	681209.657	599738.033	574197.139	497596.896	473398.777	444544.366	418148.524	495040.717
275	N,N,N'-Trimethyl-1,4-phenylenediamine	47196.219	45343.8425	43491.466	nd	nd	nd	41745.97	41428.31	41587.14	nd	nd	nd
276	N-Methyl-N-methoxy-5,6,7,8-tetrahydro-1-naphtamide	64272.057	63052.63	60861.341	66946.579	61443.157	64194.868	55645.854	50387.358	48566	47309.181	44076.414	45692.7975
277	2-Methylbenzyl p-toluate	525690.099	509352.7775	493015.456	351886.504	396075.311	349256.447	400108.264	374278.763	395338.943	444652.483	448339.989	559427.638
278	1,5-Cyclodecadiene, 1,5-dimethyl-8-(1-methylethenyl)-, [S-(Z,E)]-	744571.105	833483.302	789027.2035	486196.401	483121.936	403847.811	488273.163	458325.976	477161.021	592916.518	564685.44	578800.979
279	Acetic acid, 2-phenylethyl ester	1014757.33	1182397.02	846731.037	590738.498	582841.094	561472.972	610978.665	563836.247	572840.273	795608.923	787879.108	991769.821
280	Phenol, 2-methyl-	314554.774	275828.606	295191.69	172197.3525	190434.272	153960.433	179096.958	144713.514	154620.134	157436.207	155919.251	156677.729
281	Phenol	2866644.24	2923677.2	2895160.72	2728826.18	2668736.45	2354263.46	2443730.87	2170101.24	2232265.52	1542973.91	1570152.98	1913221.8
282	(E)-4,8-Dimethylnona-1,3,7-triene	347620.649	298525.17	249429.691	185032.9685	183736.589	186329.348	186278.436	179545.048	193011.824	187428.313	256557.2075	221992.7603
283	1,4-Methanonaphthalen-9-ol, 1,2,3,4-tetrahydro-, acetate, syn-	28428.144	28185.9695	27943.795	29542.535	26498.931	28020.733	23457.028	19158.933	23230.888	28680.724	30703.312	38851.071
284	2-Butenal, 2-methyl-4-(2,6,6-trimethyl-1-cyclohexen-1-yl)-	13949.543	12134.3585	10319.174	nd	nd	nd	11676.96	12786.989	11784.44	nd	nd	nd
285	Propan-2-one, 1-(4-isopropoxy-3-methoxyphenyl)-	129702.649	111479.7905	93256.932	43602.2525	48244.934	38959.571	nd	nd	nd	nd	nd	nd
286	2-Benzylidenecyclohexanol	102359.16	111836.833	107097.9965	60990.7935	70073.132	51908.455	76272.254	60722.786	68497.52	50915.828	48889.301	49902.5645
287	2,3-Dimethyl-undec-1-en-3-ol	348451.617	421892.644	385172.1305	266137.139	292601.068	239673.21	310346.565	255277.721	271878.724	251055.423	233996.699	242526.061
288	dl-Menthol	84356.436	83357.1865	82357.937	111310.909	95783.4425	80255.976	83222.127	78010.126	85976.099	144553.973	89561.885	117057.929

289	1,7-Octanediol, 3,7-dimethyl-	24658.154	28676.296	20640.012	nd	nd	nd	25947.707	26882.8395	27817.972	nd	nd	nd
290	Furan, 2-pentyl-	1572139.45	1946598.96	1759369.205	581426.096	632822.577	602806.533	541848.4675	529172.659	554524.276	559827.709	577981.156	568904.4325
291	Bicyclo[3.1.1]hept-2-ene-2-methanol, 6,6-dimethyl-	146444.861	143861.21	145153.0355	72102.035	72089.963	60159.551	66451.324	53869.332	58536.782	70845.554	72644.394	87106.175
292	Tricyclo[4.3.1.0(3,8)]dec-4-en-10-benzyl ether	33669.451	34436.484	26227.679	nd	nd	nd	32683.282	25372.642	26123.272	46377.476	45525.981	45951.7285
293	1-Butanol, 3-methyl-, nitrate	185827.381	167388.651	148949.921	73095.384	85982.9435	98870.503	119121.8895	99390.323	138853.456	52271.04	38400.947	45335.9935
294	Ethanone, 1-(3-hydroxy-4-methoxyphenyl)-	392288.22	391131.875	391710.0475	229628.671	207122.53	190114.523	171600.013	158233.006	146031.591	136704.666	132091.996	145593.521
295	Bicyclo[3.2.2]non-6-ene-6-carboxylic acid, 8-(3,4-dimethoxyphenyl)-9-methyl-3-oxa-, methyl ester	37557.141	38531.3775	39505.614	40698.866	39091.881	35503.521	48664.398	42508.6155	36352.833	32118.355	30734.754	31426.5545
296	4,7,7-Trimethylbicyclo[4.1.0]hept-3-en-2-one	67258.429	67030.395	66802.361	59270.044	54583.758	50929.122	51440.044	46810.094	49125.069	50591.64	51328.029	50959.8345
297	(6-Hydroxymethyl-2,3-dimethylphenyl)methanol	43612.174	52816.334	48214.254	69005.965	80176.952	57834.978	49116.984	44587.85	53976.484	74914.398	79034.891	76974.6445
298	Geraniol	52630996.9	59167048.5	55899022.7	35180427.7	36796406.1	37544221.2	40428126.1	35661577.5	35890611.9	37846989	38417444	45208425.5
299	Epicubenol	201494.939	214676.193	170338.649	155511.646	152864.197	162502.458	163042.286	154607.664	146561.235	141717.467	126669.581	134193.524
300	p-Mentha-1,8-dien-7-ol	127273.21	133761.377	130517.2935	108346.386	103414.504	82827.086	103502.473	85204.093	91556.046	81224.276	81713.497	81468.8865
301	Butanamide, N-(3-methylphenyl)-	20821.57	21489.4255	22157.281	nd	nd	nd	nd	nd	nd	nd	nd	nd
302	2-Cyclohexen-1-ol, 3,5,5-trimethyl-	145901.675	174879.822	160390.7485	111564.401	106346.875	90061.52	86614.1585	83547.133	89681.184	97441.962	96672.873	97057.4175
303	5-Methyl-2-phenyl-2-hexenal	202827.189	181878.0835	160928.978	105468.948	102734.369	103791.177	203894.153	171628.684	180193.631	171436.393	177236.855	174336.624
304	Cyclohexanone, 5-methyl-2-(1-methylethyl)-, trans-	2775542.93	2669185.335	2562827.74	1471062.9	2018588.24	1905920.08	2022269.78	2155858.16	2115640.07	3126224.79	2193929.69	2660077.24
305	p-Cresol	217951.432	222318.538	220134.985	155292.82	138126.869	118988.163	164391.17	146945.337	129499.504	123660.169	132166.328	127913.2485
306	Isopentyl hexanoate	1932298.96	1662828.01	1393357.06	918402.044	853613.6835	788825.323	nd	nd	nd	1526013.04	1053893.03	1289953.035

307	2-(1-Methylcyclopentyloxy)-tetrahydropyran	330442.052	291475.027	252508.002	174325.38	165909.467	162293.077	131167.563	132529.046	129806.08	146406.675	167238.604	209555.038
308	Hexanoic acid, 3,7-dimethyl-2,6-octadienyl ester, (E)-	142832.0185	156390.315	129273.722	68943.573	78302.441	71893.396	85548.478	64864.291	84277.925	62250.419	58222.779	60236.599
309	Fragranyl isobutyrate	126796.703	116472.711	121634.707	nd	nd	nd	nd	nd	nd	nd	nd	nd
310	p-Cymen-7-ol	1059167.46	940356.546	821545.632	749839.904	754529.817	696367.522	901529.839	740563.117	774745.766	666049.059	664386.193	665217.626
311	1H-Indene-4-carboxylic acid, 2,3-dihydro-1,1-dimethyl-	88348.763	87570.41	78569.501	70410.217	68395.714	69402.9655	71608.782	64861.317	78356.247	120339.6	132070.612	169115.303
312	(E)-5-((1R,3R,6S)-2,3-Dimethyltricyclo[2.2.1.02,6]heptan-3-yl)-2-methylpent-2-enal	231663.39	228453.861	225244.332	213250.183	205640.462	214227.963	213151.268	227130.578	207258.748	267888.21	244615.418	321686.966
313	3,5-Octadien-2-one	815014.205	887808.538	851411.3715	500159.969	536245.867	475029.524	469684.468	424271.782	441739.165	650085.702	630826.203	792953.181
314	2-Methylbenzyl benzoate	203129.262	191583.6485	180038.035	155579.212	156490.788	148970.854	146142.006	129504.868	131319.771	162085.8	168725.83	207703.491
315	cis-p-Mentha-2,8-dien-1-ol	861070.797	1030389.77	945730.2835	595297.924	596168.806	545586.697	542351.949	490188.012	512281.117	786741.233	838265.708	959002.274
316	1,4-Benzenedicarboxaldehyde	145235.683	147560.133	146397.908	89866.902	93689.669	86044.135	90771.147	81191.842	69676.165	82203.887	77573.694	79888.7905
317	Indole-3-carboxaldehyde, 1-(4-fluorobenzyl)-2-methyl-	24269.381	21455.892	18642.403	nd	nd	nd	23125.532	22007.618	20889.704	19320.549	16408.886	17864.7175
318	Valeric acid, 4-nitrophenyl ester	62480.1735	67048.777	57911.57	30498.232	36340.013	42181.794	nd	nd	nd	nd	nd	nd
319	Cyclohexanone, 4-hydroxy-4-methyl-	1413088.81	1396761.16	1265188.64	814142.069	1104979.91	892822.726	979376.429	956492.053	1034022.08	839481.68	834537.354	942524.154
320	2-Heptanone	126943.838	97647.233	109689.99	69867.16	79475.932	80339.486	82384.6765	87284.438	77484.915	138389.028	123879.148	109929.188
321	2-Pentadecanone, 6,10,14-trimethyl-	328875.625	361209.747	296395.472	376666.193	391909.529	458389.995	367899.558	423712.484	383272.395	442719.905	400825.965	485390.374
322	2-Cyclopenten-1-one, 3-methyl-2-(1,3-pentadienyl)-, (E,Z)-	165639.104	142808.3635	119977.623	129053.798	119292.33	108355.145	136470.647	109655.444	121589.628	98002.504	102757.037	100379.7705
323	Pivalic acid, 2-methylpropyl ester	113368.31	103357.446	93346.582	nd	nd	nd	99099.677	83121.311	91110.494	nd	nd	nd
324	5-Hepten-2-one, 6-methyl-	3275369.15	3095763.595	2916158.04	1961090.19	2281321.05	2189919.02	2564195.06	2474591.66	2514403.78	3506224.94	3230284.59	3948704.18
325	1,3-Benzenediol, 5-pentyl-	2794188.3	2554197.46	2314206.62	2937949.3	2668806.2	2503364.98	3386653.67	3085854.55	3061410.21	2397366.85	2203888.39	2300627.62

326	2,2-Dipropyl-N-ethylpiperidine	53812.787	53735.0745	53657.362	44635.053	51306.937	47970.995	42723.015	37314.319	39817.673	35688.02	41559.456	38623.738
327	3-(2-Isopropyl-5-methylphenyl)-2-methylpropionic acid	114844.139	112915.458	89773.204	65494.182	63286.344	51218.783	92335.563	70849.167	77217.928	125601.483	141086.109	161704.373
328	Formamide, N-phenyl-	168472.157	159512.5495	150552.942	121949.947	130397.235	112656.915	158571.515	134645.381	152212.999	86501.711	80162.327	83332.019
329	1H-2-Benzopyran-1-one, 3,4-dihydro-8-hydroxy-3-methyl-	408242.028	391250.631	399746.3295	266701.581	226551.101	209386.113	187390.68	164920.093	153716.028	149394.07	148933.383	154104.749
330	(1R,4S,9aS)-1-Methyl-4-((Z)-pent-2-en-4-yn-1-yl)octahydro-1H-quinolizine	386284.262	376277.114	340783.133	288398.395	280981.356	229130.693	361763.214	286197.898	313396.884	361942.269	377522.62	369732.4445
331	Indolizine, 3-methyl-	37630.166	45489.851	41560.0085	nd	nd	nd	57343.52	53830.676	55561.704	47816.419	50676.643	49246.531
332	3,4-Methylpropylsuccinimide	58604.989	69206.47	52491.198	56266.5825	54311.495	58221.67	61280.819	59092.141	61139.335	55746.419	54289.637	67754.195
333	3-(2,6,6-Trimethyl-cyclohex-1-enyl)-propionic acid, methyl ester	nd	nd	nd	115907.074	102801.087	109354.0805	nd	nd	nd	69437.093	77879.908	73658.5005
334	8-Hexadecenal, 14-methyl-, (Z)-	161146.667	167797.653	149761.86	401866.883	359960.513	443773.253	380634.862	475309.01	436762.934	518573.715	418774.256	528373.074
335	4-Hexenoic acid	18024176.7	18886439.5	18455308.1	9159626.78	12634167.4	11474998.6	14692769.9	12110901.5	13494096.2	11625165.6	10262684.8	10943925.2
336	Methacrolein	194960.629	176493.6035	158026.578	166627.21	229546.088	197765.54	210723.698	229610.454	225239.858	197626.871	167353.705	220514.46
337	Hexadecanoic acid, methyl ester	470374.636	471314.447	360605.066	322325.921	283522.63	273271.73	269982.966	237099.833	225379.736	194675.596	185174.443	238786.493
338	Pentadecanoic acid, 14-methyl-, methyl ester	450211.489	405330.369	380078.552	229151.94	258887.9625	288623.985	243381.411	263707.44	225200.984	511633.428	384965.048	448299.238
339	1-(7-Hydroxy-1,6,6-trimethyl-10-oxatricyclo[5.2.1.0(2,4)]dec-9-yl)ethanone	59779.858	69212.144	64496.001	36517.0215	35755.171	37278.872	70264.248	63030.242	60155.038	108793.312	99242.973	130664.304
340	2-Phenyl-2,4-octadienol	26488.002	25355.836	21661.681	15267.637	14963.603	15072.941	16457.923	15501.307	15979.615	17227.979	16672.501	16950.24
341	Benzo[b]thiophene, 2-ethyl-	32442.449	33128.973	29531.543	28883.472	29002.651	28265.366	28560.289	24753.884	25848.724	24121.666	23546.982	31570.579
342	3-Buten-2-ol, 4-(2,6,6-trimethyl-2-cyclohexen-1-yl)-, (3E)-	49883.768	55012.276	44181.31	42223.417	31569.094	35897.272	37369.8015	38202.015	36537.588	35217.419	30978.247	33097.833
343	2H-Pyran-2-one, tetrahydro-6-(2-pentenyl)-, (Z)-	1032792.42	948722.728	864653.036	817600.617	744340.497	677380.899	1002943.31	782512.112	825242.58	653955.955	620690.724	637323.3395

344	Maltol	nd	nd	nd	nd	nd	nd	10144.9455	11761.757	8528.134	24453.246	28957.403	26705.3245
345	Carbonic acid, tridecyl vinyl ester	41362.272	42544.0555	43725.839	44214.526	46779.213	34245.85	44253.715	34135.782	39194.7485	30065.27	25795.264	33937.068
346	4-Isopropyl-1,6-dimethyl-1,2,3,4-tetrahydronaphthalene	112772.1	119887.3335	127002.567	138348.765	139713.08	139030.9225	69528.827	60064.353	61488.28	68616.214	58309.853	82654.568
347	2,6,10-Dodecatrien-1-ol, 3,7,11-trimethyl-	32989.862	40996.502	36993.182	16890.563	14679.812	15785.1875	nd	nd	nd	nd	nd	nd
348	2-Cyclohexen-1-one, 4-(3-hydroxy-1-butenyl)-3,5,5-trimethyl-	143171.257	121856.169	100541.081	nd	nd	nd	43903.587	37907.825	31912.063	nd	nd	nd
349	2,6-Octadien-1-ol, 3,7-dimethyl-, (Z)-	12070936.2	10427402.24	8783868.28	5934448	6456974.3	6624487.18	7074361.28	6015757.91	6095314.95	7068473.38	7120420.62	8835884.76
350	Azulene, 1,4-dimethyl-7-(1-methylethyl)-	188670.716	188803.827	188737.2715	93312.265	102052.043	84572.487	64629.597	68541.189	60718.005	56325.386	54453.437	58261.136
351	Hexahydro-3-butylphthalide	43624.004	47798.9395	51973.875	67137.1	62452.566	64794.833	40304.504	37693.519	35082.534	31356.828	35238.058	33852.864
352	2,4-Di-tert-butylphenol	5079368.68	5651209.83	4968279.86	6617652.56	6231442.6	7003862.52	11284511.2	9676189.88	8740794.95	8049714.03	9678149.55	8863931.79
353	Farnesol, acetate	600474.37	524518.646	448562.922	417433.032	375374.995	316341.869	304271.6995	302321.211	306222.188	319886.347	434331.2105	377108.7788
354	Triethylene glycol	148296.106	132707.558	130748.061	130890.601	119610.658	131516.387	121152.65	126286.523	101900.731	114366.06	103695.226	118903.623
355	Methyl jasmonate	199475.293	221975.315	173471.363	178159.895	176944.852	207063.036	186376.903	232478.351	195908.162	232446.606	208571.876	267921.884
356	2-Pentenal, (E)-	3353398.95	3285954.23	3218509.51	2503853.52	2780551.88	2698877.72	2233921.49	2571341.76	2572284.59	2340289.55	2184770.44	2391246.24
357	Platambin	26180.127	24332.698	25256.4125	18874.297	19840.038	16094.939	nd	nd	nd	nd	nd	nd
358	2-Methyl-4-pentenoic acid	290606.01	264991.2405	239376.471	133795.679	136410.7275	139025.776	nd	nd	nd	nd	nd	nd
359	2,4,6-Octatriene, 2,6-dimethyl-, (E,Z)-	245321.473	290267.1955	335212.918	249541.07	255143.715	241399.43	177363.073	201377.175	230166.643	211815.942	226337.863	231627.68
360	8-Hydroxy-2,6-dimethylocta-2,6-dienoic acid, ethyl ester	58045.103	51979.0935	45913.084	56815.187	45626.97	42297.044	49402.763	50926.562	47878.964	35415.201	32968.952	34192.0765
361	3-Buten-2-one, 4-(2,6,6-trimethyl-1-cyclohexen-1-yl)-	11028001.5	9549464.425	8070927.35	5722166.72	6472572.37	6762892.09	8545570.49	7586116.54	7463912.65	6552809.81	5990072.44	6271441.125
362	3,3-Diethyl-pyrrolidine-2,4-dione	475735.615	429670.81	452703.2125	556636.043	465782.85	574037.381	640314.162	648415.798	607222.261	678843.572	775611.185	727227.3785
363	Benzoic acid, 2-hydroxy-4-methoxy-6-methyl-, methyl ester	1283743.76	1321135.06	1061014.06	984048.647	837018.598	793180.067	724169.89	651151.809	611282.566	607045.519	578906.254	635024.622
364	Isoaromadendrene epoxide	73237.472	66519.957	56007.408	48651.623	44019.888	44883.51	40034.593	30673.131	35353.862	10124.4135	9930.65	10318.177

365	4a,7a-Epoxy-5H-cyclopenta[a]cyclopropa[f]cycloundecen-4(1H)-one, 2,7,10,11-tetrakis(acetyloxy)decahydro-8,9-dihydroxy-1,1,3,6,9-pentamethyl-	88948.697	96243.595	71722.894	77136.587	63085.081	59692.51	77214.225	64782.09	69911.768	46637.155	45150.905	45894.03
366	Benzaldehyde, 3-methyl-	1454016.36	1292578.33	1352374.1	1107167.48	1197533.29	896871.192	1114158.68	930190.001	1056995.71	1114074.56	1205534.39	1463472.99
367	Isoelemicin	190905.478	194750.973	192828.2255	nd	nd	nd	90720.042	80212.704	78434.08	70097.879	67380.76	71615.112
368	(-)-Myrtenol	3076959.74	3311444.54	3194202.14	1636524.75	1795688.01	1791034.95	1765342.46	1609246.39	1609628.4	1888939.34	1913032.23	2189795.24
369	Quinoxaline-6-carboxylic acid,(1-oxo-1,3-dihydroisobenzofuran-5-yl)amide	51348.236	55457.296	43589.78	41603.136	35810.73	38706.933	58125.586	50921.395	47752.596	40648.367	39976.114	40312.2405
370	Coumarin	1244577.22	1415414.21	1132036.26	1173586	1038869.43	1009702.08	1179779.34	1033003.68	1082424.64	946409.14	926276.646	1274003.19
371	Furan, 2-ethyl-5-methyl-	26474.415	23755.849	21037.283	21052.519	26426.49	23739.5045	19961.5635	16885.748	23037.379	nd	nd	nd
372	n-Butyl cinnamate	21909.365	26613.852	20323.844	16036.073	14597.954	13159.835	14473.782	14453.756	14493.808	15005.01	13926.802	16419.311
373	3-(4-Amino-phenyl)-2-(toluene-4-sulfonylamino)-propionic acid	125332.092	148018.907	170705.722	61782.702	63310.662	60254.742	91237.0255	80636.741	101837.31	77636.631	56136.473	66886.552
374	1H-Indene-1,3(2H)-dione, 2-(2-methylbutylidene)-	39205.564	36203.967	37704.7655	nd	nd	nd	nd	nd	nd	nd	nd	nd
375	Benzoic acid, nonadecyl ester	11008.93	13245.512	12795.057	nd	nd	nd	20225.24	20335.959	20114.521	18732.671	16175.105	21290.237
376	3-Octanol	708206.832	814042.724	761124.778	432209.004	578062.899	569141.947	633677.388	694747.1	658338.534	923029.379	712666.444	817847.9115
377	3-Methylene-1-oxa-spiro[4.5]decan-2-one	72889.846	78285.338	75587.592	55223.008	47808.3485	40393.689	56943.354	43972.973	46686.371	43900.848	25996.416	34948.632
378	9,12-Octadecadienoic acid (Z,Z)-	49534.832	45319.316	42667.875	31880.13	28626.936	27422.96	26192.505	23713.261	23881.009	41455.737	38604.2	52261.362
379	1-Isopropenyl-3,3-dimethyl-5-(3-methyl-1-oxo-2-butenyl)cyclopentane	299232.52	345689.267	273222.386	299355.635	239526.726	264374.268	312807.051	260906.348	248766.136	234310.954	211211.773	222761.3635
380	Cyclohexanol, 3,3-dimethyl-	310016.616	345989.601	328003.1085	161447.46	172711.009	183974.558	207163.406	174249.981	192671.335	198444.202	125049.607	161746.9045
381	3-Methoxy-5-methylphenol	233683.507	229880.798	180814.04	157957.911	150574.715	132397.517	131477.135	117987.693	107835.419	109140.151	99884.6	110602.689

382	7-Acetyl-2-hydroxy-2-methyl-5-isopropylbicyclo[4.3.0]nonane	43245.597	44408.811	35802.629	36052.254	30939.241	29506.931	25542.871	21839.517	20562.821	20283.045	19164.724	19723.8845
383	Phthalic acid, hept-4-yl isobutyl ester	1277183.69	1462787.27	1210641.99	1835468.01	1762668.94	1781079.38	1887760.69	1752414.13	1816601.46	1730720.1	1692128.98	1878922.78
384	1-Hexadecyn-3-ol, 3,7,11,15-tetramethyl-	41426.599	55660.411	48543.505	74242.127	59375.049	70021.651	81215.733	77704.556	79699.128	75682.647	69028.23	94055.997
385	Vanillin	1938539.02	1517019.41	1727779.215	nd	nd	nd	nd	nd	nd	nd	nd	nd
386	3-Methoxy-4-[3-oxo-3-(pyrrolidin-1-yl)propoxy]benzaldehyde	74212.431	75142.179	73277.148	79404.699	80478.761	97518.416	80742.933	74376.79	79285.06	78815.064	79520.045	75208.332
387	9,12,15-Octadecatrienoic acid, methyl ester, (Z,Z,Z)-	115171.79	100886.396	90206.601	66933.43	50235.671	63350.027	55036.712	57892.438	62231.287	100651.137	84041.67	109620.196
388	(2R,5R)-2-Methyl-5-(prop-1-en-2-yl)-2-vinyltetrahydrofuran	1102350.4	939578.824	949963.667	642316.496	845027.018	667829.998	660009.669	604004.622	655085.507	524384.448	475335.458	620438.806
389	Xanthoxylin	108566.002	115827.626	90754.242	89685.454	79806.441	74483.949	68443.104	58034.357	56253.324	56186.037	57020.328	59886.585
390	Phthalic acid, butyl hex-2-yn-4-yl ester	181055.474	194307.97	169884.974	233055.2	201588.879	216349.635	221105.145	212093.871	210835.531	190718.257	187545.572	253133.32
391	Phytol	1502648.57	1510881.88	1520107.88	2256180.83	2011773.46	1767366.09	1769813.17	1509985.31	1452575.22	1215194.6	1116453.7	1488188.82
392	2(4H)-Benzofuranone, 5,6,7,7a-tetrahydro-4,4,7a-trimethyl-, (R)-	2290678.02	2566547.48	2428612.75	1804091.98	1693591.76	1682681.84	2102426.08	1956647.1	1956991.82	1738281.84	1548603.98	1643442.91
393	2,6-Octadienoic acid, 8-acetoxy-2,6-dimethyl-, methyl ester	80460.405	99230.805	89845.605	83675.453	64513.666	67399.783	88286.954	75210.151	81113.417	70820.831	70656.853	97734.371
394	Caryophyllene oxide	63631.574	55246.263	46860.952	56903.557	51472.602	54188.0795	50358.936	47887.561	42019.841	43168.845	45016.68	44092.7625
395	2-Octen-1-ol, (E)-	438592.548	380816.425	323040.302	290045.2	389570.908	333739.526	400373.333	485372.26	470761.509	459416.138	455406.277	457411.2075
396	(2R,5S)-2-Methyl-5-(prop-1-en-2-yl)-2-vinyltetrahydrofuran	330143.065	286793.154	276588.429	187800.018	248582.917	199718.559	205348.897	177981.886	190613.219	156677.259	149572.752	192475.486
397	2-Butanone, 4-(2,6,6-trimethyl-1-cyclohexen-1-yl)-	79186.07	92807.547	78938.363	90300.38	69281.715	72713.027	74802.114	67766.314	67953.271	60361.717	67387.102	83196.919
398	2'-Hydroxyacetophenone, TMS derivative	114649.095	128237.106	107842.158	133279.159	107502.315	113201.768	120058.747	113039.336	113843.028	105689.414	107295.436	130207.534

399	Propanamide, N-ethyl-	nd	nd	nd	nd	nd	nd	67111.779	49590.51	58351.1445	nd	nd	nd
400	Tetraethylene glycol	171724.517	169097.2185	166469.92	144250.529	139324.151	141787.34	158311.053	128793.035	128638.494	109580.466	114803.987	106484.442
401	7-(2-Hydroxypropan-2-yl)-1,4a-dimethyldecahydronaphthalen-1-ol	51026.058	46542.107	43348.967	44203.187	37080.061	36763.714	35160.857	32385.262	29943.442	28104.799	26661.192	29450.447
402	5,6-Dimethyl-2-benzimidazolinone	73749.032	78494.44	61234.263	60943.901	53509.663	52949.409	64829.072	56469.074	59850.761	65975.349	66238.358	88143.258
403	4-Benzyloxybenzoic acid	134130.399	145274.252	139702.3255	nd	nd	nd	111885.134	102720.305	96192.59	88308.95	90930.795	110758.16
404	Naphthalene, 1,2,3,5,6,8a-hexahydro-4,7-dimethyl-1-(1-methylethyl)-, (1S-cis)-	2136928.95	1856142.025	1575355.1	961906.6195	1017311.14	906502.099	1057723.22	867804.148	795961.353	719893.163	596567.306	658230.2345
405	Benzaldehyde, 3-ethyl-	266865.37	305243.656	286054.513	160085.042	173004.641	179319.218	208133.548	204464.789	195119.254	243674.165	233735.828	286354.123
406	Dodeca-1,6-dien-12-ol, 6,10-dimethyl-	217024.672	242677.094	229850.883	133129.361	156568.71	115841.432	156636.042	132596.907	151683.804	136972.38	108299.638	165645.122
407	Adipic acid, 2-ethylhexyl isohexyl ester	25210.317	25700.41	26190.503	nd	nd	nd	nd	nd	nd	35036.183	28877.745	41194.621
408	Benzaldehyde, 2-hydroxy-5-methoxy-	45286.881	54183.546	42443.93	36079.798	30648.278	41511.318	47961.542	42969.263	37976.984	37607.682	35037.741	47171.59
409	2-Hydroxy-4,4,6-trimethylcyclohexa-2,5-dienone	97422.765	114037.757	91121.092	115838.249	86353.572	96560.906	97763.365	88597.122	94744.207	87752.061	81988.698	107955.912
410	2H-1-Benzopyran-2-one, 7-methoxy-	74387.198	81624.649	74398.442	87536.322	68578.487	76056.885	71462.759	72583.864	78150.478	74698.874	71745.988	86041.307
411	3,6-Octadien-1-ol, 3,7-dimethyl-, (Z)-	814772.329	708300.1615	601827.994	413097.373	447383.91	474295.205	479338.126	408117.77	417861.294	497415.15	501114.698	607096.196

* nd: The compound was not detected.

Table S2. List of the nonvolatile compounds in the black tea samples.

No.	Compounds	Peak area											
		**CRSD-1**	**CRSD-2**	**CRSD-3**	**RLSD-1**	**RLSD-2**	**RLSD-3**	**CLSD-1**	**CLSD-2**	**CLSD-3**	**HASD-1**	**HASD-2**	**HASD-3**
1	Uridine 5'-diphosphate	7518558	7816046	7432295	7261215	7118593	6897535	10395091	9547565	9971328	9116428	9409367	9702306
2	Epicatechin isomer	4275194	6979665	5627429.5	5792711	5514084	6071338	9592271	10455508	8833676	3958839.5	3958265	3959414
3	Gallocatechin	16641814	20183373	18412593.5	14641763	13936690.5	13231618	17714163	15787641	15981555	15469364	16149127	17834242
4	Erythrose	15885699	15438558	14617360	14475986	15868932	15172459	15414367	14658556	16430409	12406152	12344092	10477863
5	Phytosphingosine	160826026	177671561.5	194517097	166127767.5	175236376	157019159	164721175	151526931	190816709	185390234	180717740.5	176045247
6	Quercetin triglucoside isomer 1	710803	670307	733843	618952	520793	569872.5	582453	560924	480454	695464	797971	746717.5
7	Caffeic acid	1419435.5	1289311	1549560	964539	1025854.5	1087170	1117976	1207083	1570392	1462200	1638778	1710357
8	Methylhistidine	3561141	3030171	3295656	3285148	3361973	3208323	2835134	3181981	2781757	3155564	3258931	3207247.5
9	Raffinose	94811918	87540688	93211644	96121564	97550964	94188107	93261423	89685768	97774008	81563826.5	88799959	74327694
10	Salicylic acid	6276220	6311032	6214810	4912377	5251006	4952503	5567260	5304057	4791887	5911367	5878863	5846359
11	Caffeoylquinic acid 4	450888	426612	475164	364875	352626	358750.5	472827	514091	532102	518328	579946	456710
12	Gamma-Aminobutyric acid	80710690	82381661	85214964	109245172	108315629	108614876	65849150	77531250	73988262	80737622	77144402	84330842
13	Oxidized glutathione	52270871	50716283	52617431	49590786	48592686	48074032	47135044	45086539	47263817	48537759.5	46042745	51032774
14	Val-Pro	21442166.5	22975963	19908370	21042516	20575442	20617284	25490442	19596539	22107340	22422605	22908542	22665573.5
15	Citraconic acid	4698348	4364323	4899416	5492711	4928696	4810891	5196715	4484385	4860718	4944380	4985799	5027218
16	1-Pyrroline-5-carboxylic acid	26108522	24321323	25214922.5	22633620	20375978	19302345	22272621	23251048	22600903	21586907	21511405	23119659
17	L-Fucose/Rhamnulose	7872249	8300754	7818764	8150360	8050159	8143864	7369693	6877905	7230115	6316514.5	6024974	6608055
18	Citric acid/Isocitric acid	24106535	25298873	24444388	13343822	12364027	14323617	19788466	22610991	18547656	19250031	19572846	18927216
19	Leu-Pro/Ile-Pro	338934	318116.5	297299	297953.5	260496	335411	326265	287105	306685	191583	177860	205306
20	Quinic acid	9938598493	9189742029	8720250428	9786036822	9978697707	10168548184	10906660394	10715852824	9704900823	9413498343	9977307444	8849689242
21	Vanilpyruvic acid	17480424	18526602	19572780	20215024	21893247.5	23571471	20970977	24923893	20999122	20187700	20918648	21649596
22	L-Methionine	6052739	5945544	5892966	6690405	6098656	6394530.5	5056898	5826778	4301816	5638404	5698450	6189327

23	Ser-Val/Val-Ser	33283206	33737277	32716019	29488893	28721332	30536854	23915063	28844797	30944605	23843688	28163259	28508562
24	Cyanidin 3-arabinoside	21049923	20591872	19882764	21200699	20879915	21521483	21821439	20692376	20528999	22304171	21675887	21750192
25	L-Proline	1130249532	1201912216	1133600062	1131961623	1096728798	1065461019	1117132275	1092096124	1075899440	1079668247	1072472332	1256993014
26	Caffeoylquinic acid 1	2939860	2962439	2893222	2991769	2872877	3263133	3576843	3779504	3253269	3958052	3303362	3544552
27	N-Acetylvaline	239326848	217434117	239712091	219736873	213416256	228563052	214555993	218002245	221346289	230688616	219533755	258427643
28	Assamicain A	506471339	503986004	458435721	347668999	367057015	381173029	324938765	323884329	336487715	471242393	551789190	542656523
29	N-Acetyl-L-alanine	19455512	16866486	18009045	16433177	16455544	18441442	15773578	19621302	18577168	17947110	20509542	17724605
30	Adenine	1154828746	1136446353	1203007860	1122662293	1144654573	1186817685	1090886211	1195296253	1205741732	1185026496	1081655545	1276461136
31	Gallocatechin gallate	5779036	6072527	6366018	16306470	16527247.5	16748025	20727320	21500629	21113974.5	6279033	6167020	6363932
32	Adenosine monophosphate	72663297	78488381	68922464	71707639	68649129	66781705	71670878	78444986	81440538	96629322	86766095	83631548
33	Deoxyribose	15559168	16041832	15374101	15990925	15831114	16150736	16328466	17078973	14270802	15079617	15585677	15320263
34	L-Carnitine	23017426	24997175	22646900	22482105	21548842	24298471	22783075	21508854	22746999	24120244	22801699	26239992
35	N-Acetyl-L-aspartic acid	8311810	9191136	9667073	7951102	8464867	8006070	9370522	8400036	8453010	11559463	11717852	13068607
36	Camelliaside C isomer 1	12717583	12868561	13158582	12954127	14264530	13838444	14031213	13798804	13347089	14957126	13070713	13959083
37	Epicatechin-(4beta->8)-epicatechin-3-O-gallate	6487748	6739128	7093549	5585522	5825819	5694358	5406810	5762073	5434745	7348117	6972339	6428821
38	Guanidinobutanoic acid	23046904	22537398	23943148	23106203	21794680	22450441.5	19811755	27059785	25956761	16875792	18170237	17523014.5
39	Oxoglutaric acid	15087499	15068948	16960179	14734605	16038176	16499549	15584314	15902518	14901195	15025405	15422814	17013593
40	Succinylacetone	22892679	24288927	23636741	22257390	23775525	24214320	22093273	20901520	21533726	17203128	18844487	16598085
41	4-Phenylbutanic acid-O-sulfate	4169905	4031336	4253404	3826568	4203059	4411949	4060493	3873747	3868254	4463835	4097526	3950882
42	3-Dehydroquinate	18956450	18626770	17488063	15679195	15409529	15651509	15557443	16292657	15912423	17354334	15359173	16814121
43	Ala-Ala	151104345	160787437	144548632	150907276	145853904	143878280	136039334	130154111	132221086	130076722	134399173	145981528
44	Indole	3012701	2654580	2833640.5	2570797	2624269	2787614	2961317	3083560	3205803	2877446	2924100	2830792
45	Dihydroxybenzeneacetic acid	2296045	2119905	2207975	1623570	1739252.5	1854935	1471413	1699576	1305493	1923211	2029800	1976505.5
46	ADP	25456937	25437124	21574891	28546299	24849508	26702278	31888666	26393567	26407863	26601539	23975481	24253595
47	1-Methyladenine	40245123	41465411	41696546	44015911	39665181	44891440	41194557	38088084	40455631	41502261	43861601	46521199

48	Theasinensin A	15089051	16505989	15797520	13392413	14779490	16166567	8556629	9364268	10171907	13433656	17066332	15249994
49	Procyanidin B-isomer2	33333507	29698062	31309191	22827805	27361574	26087706	25405302	25569253	24191062	29745210	31847434	28522962
50	Ala-Thr	169743168	174894457	160151924	176105721	166757574	169923003	140967778	142182817	140465627	131998772	135872362	146829801
51	O-Phosphoethanolamine	19517154	19643643	19525323	18265586	19258463	18540227	17224281	17388667	17524689	16340654	17762989	18192687
52	Pro-Val	14958655	15667424	15506494	18097544	17964216	16924885	16535995	16454228	16353282	16428053	15646832	17472980
53	L-Valine	823215403	845212410	828427911	829454901	814968970	817172866	808904149	833975824	775719137	816067654	737290139	807379620
54	Disaccharide (Maltose, Lactose)	29331504	29199884	29955984	27295283	28729524	25010597	26390592	25772987	24319845	25472021	23016914	23517937
55	Pipecolic acid	199275284	205593511	193191135	181516213	188135838	184748118	191051814	180468094	209082670	203315627	184222810	187085958
56	Glucaric acid/Galactaric acid	325613862	301384519	307259019	298570008	331327854	321087439	290951215	318885734	276832621	252557330	244745130	227490497
57	Hexose-phosphate	203369775	199902538	191044899	186166316	210995647	201774339	180150494	183057292	175705202	175317648	162203695	158731475
58	Fumaric acid	84138309	85387785	82002023	81729981	87604416	80226149	81685907	87287709	80790293	81608848	74731437	82284022
59	Trimethyl-L-lysine	55774914	57330084	53846791	51344840	55563167	50422232	50863788	56474933	55848014	51252609	55212701	56762972
60	Epicatechin 3-O-(3-O-methylgallate) isomer	648515	591987	662211	566878	622945	598628	645812	629663	637382	658922	721212	663061
61	Glutaric acid	3941610	4007290	3875930	5432754	3928016	2423278	3796100	2529696	3220336	4591523	3936796	5246250
62	Gluconic acid	813449258	789999732	780729462	739766303	877743033	829000987	701961296	728962471	696638880	619717730	593401827	562652273
63	Theasinensin F	11649002	10842264	9942343	10603217	11454592	11736569	10440471	11538065	11405660	12527032	11932502	11376496
64	N-Acetylglutamic acid	38832337	39214921	40634195	36786764	39404594	37601411	38121223	39737896	38438458	34860996	38143897	36175749
65	Epicatechin 3-O-(3-O-methylgallate)	15495146	14456242	15059509	12907369	14462373	13835640	14923132	14059514	12802365	16327275	16630240	15243727
66	L-Histidine	40158906	45674330	40430141	38575322	39516761	42059164	35889915	34381146	36448474	39920473	38132641	36476883
67	Nicotinic acid	119457727	109515324	127814375	115966910	122156542	129177414	107032745	105203275	111624452	108747146	107735966	116736944
68	Pro-Ser	102519491	107603629	105626773	107163786	102410918	108532382	108151287	105139210	102585620	102942282	95461063	95603784
69	L-Glutamic acid	1796488670	1883263771	1753090876	1848307437	1805652901	1773426117	1775069747	1715159433	1663677514	1626639550	1660170453	1762979769
70	Pyrocatechuic acid	60469472	63553301	59544327	59472645	63356780	61763522	62100412	61953300	59835681	63480520	61251273	66570137
71	Homo-L-arginine	22540074	22478314	22586552	20821918	21930595	21418540	21039280	22125694	22894682	21492513	23366132	22353970

72	Nicotinate D-ribonucleoside	174240144	168468973	179675265	160894552	156074095	165396841	144847943	157871866	166332937	168340761	160333725	173628493
73	L-Theanine	26583916321	27640648482	26156518947	26867131480	26738241877	26933260716	25378248601	26556950410	24221818792	25264533447	23407174599	24761981605
74	Glyceric acid	14387537	13516423	15373219	16374728	16188479	18256115	18074153	18995123	15526547	14904546	14079258	15179447
75	Phospho-glyceric acid	59659386	55529328	53211227	51005194	50349176	50662104	58464780	57901512	49933400	61201817	58123064	62709201
76	Ile-Ser	28534096	30632099	27427382	28716434	31587887	32122381	29588834	28082180	29354692	28948275	26953545	28545176
77	Shikimic acid	132812016	137010763	133068418	148571152	139841234	137322818	155766582	158907166	153876571	142591827	141924512	133313417
78	Isorhamnetin	3136100	2934115	2744897	2850404	2976785	2890359	3293456	3115441	2885684	3623984	3419954	3390801
79	Malvidin 3-glucoside	5174816	4994584	4968003	4898223	5687872	5314913	5501745	5193798	5393571	5277861	5516626	5136308
80	Glu-Ala	41614547	40851362	40855028	40253709	40390934	40193798	37626663	39144647	38798402	36982689	34590323	36677213
81	2-Methylcitric acid	43804906	45934671	42476821	41054447	42368260	45911758	47801889	50086580	48035726	48616181	52198360	50302201
82	Ribonic acid	504898276	495496078	510859969	470300419	548264308	494860705	456107103	492553038	459365638	399664586	407223226	380104225
83	Gluconolactone	17924557	18080126	17332681	15533073	18122300	16331556	16329685	16862126	14982668	17553282	16780497	17994756
84	Sedoheptulose-phosphate	1203483056	1092755252	1142316605	1111551684	1136950249	1144293545	1355972316	1332984493	1139716416	1171572391	1139072727	1092857463
85	2-Hydroxyglutarate	42082715	43051942	42113024	39313296	40700737	40403611	39799693	39578730	40588748	42369840	40724898	39561678
86	Quercetin triglucoside isomer 2	3416094	3371202	3536422	3385072	3305433	3100666	3494387	3142510	3204234	3690744	3506507	3461489
87	Myricetin	22106655	21036911	18968009	20868669	20937684	18933380	24654966	23640834	22712401	20728292	20481558	21828258
88	N-Acetylproline	9954242436	10509316949	9704077442	10034622369	10143501619	10436012867	10232211440	9476167242	9653046666	9757179923	9498806395	9117027140
89	Thr-Pro/Pro-Thr	40942919	41048865	43705739	33594008	34117883	35811260	30677935	35373237	35217900	32378571	32018399	34045217
90	L-Threonine	184358067	193111317	182154361	185449930	182445052	179087127	172838124	176675682	168991612	175991811	182387440	187928776
91	Pinitol	66940673	68693510	71368663	65592999	69500592	66966622	57604681	62097264	60748254	49768244	49772007	52572118
92	Methylsuccinic acid	9283715	9528134	9222388	8249134	9233529	9012755	8839685	8895293	8946805	8885028	9344117	9428537
93	Linalool primeveroside	1299200	1351309	1285102	1311360	1472034	1430997	1600460	1559890	1541455	1302675	1239955	1229910
94	Procyanidin C1	10380164	10343090	9316564	8445022	8759034	9140740	8521934	8367555	8738061	10054740	9514435	9555066
95	Leu-Ser	24784620	26040527	22863728	27641030	27840773	27690278	24945618	26228949	24270465	21696729	22318206	23078485
96	Proline betaine	147848173	147627415	147984294	139650137	138412402	135563297	125433939	152402791	147214316	154915956	148156150	157299246
97	L-Phenylalanine	3542808112	3760751131	3534046382	3471740811	3411881360	3482396109	3344036519	3407437923	3429779823	3175919941	3216794331	3362732616

98	Choline	1594192741	1703584662	1647161769	1668884601	1703875977	1691461409	1785737391	1746225008	1716579209	1770291499	1776956134	1683231083
99	Naringin	13230002	13196513	12869711	12913215	13435818	13859848	13978848	13711298	13699518	14148668	13375778	13522985
100	cis-4-Decenedioic acid	5208670	5134303	5473124	5293789	5563023	5894775	5003168	4863849	5110954	5130289	5309673	5446438
101	2-Hydroxyphenethylamine	1995433169	1974015361	1933331108	1915693624	1859762705	1881515960	1821436677	1849790897	1916562756	1754154571	1798786123	1861470847
102	Procyanidin C1 isomer	13238535	12508650	12113866	10172539	10493389	10656037	10867567	10811405	10581132	11235314	11890010	11476226
103	Uridine diphosphate glucose	86492649	76003622	77336283	73125161	77836247	81965082	68164334	70028036	72155198	67298171	64223539	67681544
104	Quercetin-3-O-galactoside	39454546	38408435	39286825	39423704	40634802	40653523	41811353	39202067	39150429	42395335	40947643	40172336
105	Nicotinamide riboside	22773472	22972437	21869157	28196663	26729316	25121335	14042172	14087316	13666000	10871159	11089295	11474246
106	Hydroxypyruvic acid	8195679	8570340	8329231	7998862	9002794	7977039	8203546	8700386	7729800	7812213	7750907	8148672
107	L-Arginine	487936290	522155164	500681567	482507680	497505084	497305118	472118310	470633713	495378362	468089193	469814955	447760083
108	Cyanidin 3-(6''-malonylglucoside)	7202820	7457500	7544532	7440312	8229703	8288454	8184656	8112804	8455935	7561501	7945830	7878947
109	Peonidin-3-glucoside	163576	148354	133132	67498	90219	78858.5	100726	113540	105267	146401	137304	141852.5
110	Glycerophosphocholine	164881639	160600366	153893905	151107570	138160266	142076051	149800730	147166016	146226550	131819321	138903821	135158722
111	Uridine 5'-monophosphate	105098101	101861177	100774876	104876131	106707565	104643883	126496102	124449863	126545398	123837103	117709541	119583072
112	Glp-Gln	162534770	157994783	154126700	148426350	133973779	137828261	146533134	145007277	147207570	127952866	133891933	133898792
113	N-Acetyl-D-glucosamine	33199808	35371130	33033753	34412980	33126155	32720722	35831370	36820609	36663628	37046791	35707968	37550697
114	N-Acetylthreonine	75866196	80132402	77325246	73730765	69921881	69788417	89401637	88084673	84718714	127261806	122170013	128038951
115	Succinyladenosine	6380484	6708051	6609995	6749802	6519999	7035476	6780305	7119923	7164178	7043274	6756077	7074476
116	Procyanidin B2	9230174	8703672	8801301	6856282	7031887	7112704	7538019	7441175	7640264	8581851	9021763	8818711
117	Ellagic acid	1256317827	1225652371	1155055198	1298502941	1323082419	1307627702	1533465734	1507982771	1557564806	1403332371	1378225068	1446865677
118	Luteolin 3'-acetylglucuronide	10710728	11323620	11548894	10015836	9691004	10309457	8737013	8771471	8962082	12281194	11700461	12088411
119	Kaempferol-3-O-galactoside	77583419	77838101	74238227	79444338	78983467	82552971	84932865	78391379	81169272	79558253	79520281	82939710
120	Catechin-(4alpha->8)-epicatechin-3-O-gallate	23249655	21350826	21484110	18279714	18232053	17845668	21213583	23818035	23062210	20080954	21002397	20225702
121	Kaempferol 3-galactosylrutinoside	318155600	316105884	307775160	327464892	324464161	330716360	347301561	321215274	329796049	319001924	314843001	329840229

122	Epigallocatechin 3-O-(3-O-methylgallate)	13488241	13476563	12911376	10012425	10535728	10855174	10266794	9487304	8514491	13327995	13821632	13945055
123	Syringic acid	1142280	1045970	1094125	989259	1074616	1159973	1074604	1038340	1167227	1034418	1021518	1047318
124	L-Asparagine	325225410	351401156	323943380	335126504	330046474	323330504	326288228	325225756	312081500	313857169	309169982	323818152
125	Catechin	56219993	55118991	54410693	49590683	47570266	48436484	55230735	54598716	52381127	55706812	58342358	56545360
126	Quercetin 3-(3R-glucosylrutinoside) isomer 1	169212442	165235109	164303401	170577358	173873096	177934475	179082643	168042310	170845738	182523118	179113763	174190453
127	Camelliaside A isomer 2	2343098811	2338444148	2276421578	2278811890	2464256861	2346447269	2475108762	2427174556	2419070673	2436658676	2329710935	2357147366
128	Catechin 3-O-gallate	3130967306	3125839821	2976210830	2855196633	2884002509	2943011262	3079659226	3059900111	3074608761	3267600168	3162080216	3125677007
129	3-Methylxanthine	74857709	72970655	69637511	74778294	73541042	75638533	82327870	86134769	86597167	78890904	81015267	82619113
130	Quercetin	157688265	152074658	145848377	144206644	147320938	153348260	149070492	143381806	140051610	153326246	160544537	156579282
131	1-O-Galloylglucose	501290441	497364967	481256606	534408475	464546723	461051642	567547453	557887785	549959175	506294851	498373813	521036581
132	Caffeoylquinic acid 3	18661448	18809000	17509464	18468948	18505241	17674371	19605694	20544604	20873429	19061704	18239113	18602821
133	Quercetin isomer	107197402	105271586	97695865	124167077	126926284	132956619	126650923	120476215	121156795	132832126	138366165	137458992
134	Theasinensin B	13238226	13186435	12370358	12605633	11372032	11919109	11811901	12867138	12852112	13435488	14018222	13817726
135	Azelaic acid	3750787	3218230	3617283	3427684	3286928	3568440	3969405	3191197	2823675	3293724	3112639	3203181.5
136	Xanthurenic acid	2945989	2750105	2739445	2765459	2765926	2932589	2940753	2899795	2890399	2988451	2894192	2879271
137	Quercetin-arabinoside	5410821	5147596	5040687	5353056	5300020	5608641	5585754	5231346	5252578	6002191	5819178	5780772
138	L-Aspartic acid	375484019	389266474	368449114	376428927	367384351	357509738	370185828	371075406	361700534	371106733	365142480	380011290
139	Kaempferol 3-O-rhamnoside-7-O-glucoside	125036054	123138025	122779931	132081229	137880585	139588366	139564313	138189911	133720250	143986587	138468625	140395191
140	Ile-Thr	54434854	57633237	56902448	53956835	51122440	59679927	54773532	55781426	51669363	54908354	53792120	52778318
141	Camelliaside C isomer 2	21716826	22131080	21127744	22231335	21750881	23144062	22653360	22177788	22382745	22627594	22071676	22941154
142	Strictinin (Corilagin)	677022452	672345239	632522749	696939351	706134990	698015938	853003826	797776009	816846749	733510229	707053108	728449783
143	L-Tyrosine	551004969	553860008	515257583	561571106	534562962	514861805	498085569	526027170	521835650	508773804	526527495	525466277
144	Linalool oxide primeveroside	24705527	24355459	22025827	21415796	22688847	21756948	25538715	22989161	22890687	21320134	21113768	20561770

145	Pyroglutamic acid	304164072	305235807	288476847	265955293	280309598	270308004	336033494	346889629	333520223	520053822	534982390	538813884
146	L-Leucine/L-Isoleucine	2914256305	2968140284	2828321097	2778918242	2820033609	2825826982	2746439167	2913952913	2735856287	2776464779	2679133043	2753782199
147	Val-Glu	592109466	569176124	517095241	512925946	504764146	513170633	464098441	491559837	465187554	500963813	519351767	506311053
148	Acetoacetic acid	17915449	17883426	17899437.5	17842492	17835415	17869440	14063340	16262385	16070024	17166014	14235275	15383028
149	Quercetin-4'-glucuronide	26638470	26163427	26189468	23037252	24746755	24389421	30731095	29932413	31066321	27319776	26529674	27454772
150	Kaempferol-3-O-rutinoside	423871239	422160488	398035503	423779354	432355302	443441790	452351014	428990951	439839044	461825750	445875473	457415019
151	Gallic acid	5450805194	5491996937	5279423122	5503896728	5920632245	5691410015	6257523595	6300399939	6352458574	6245764990	6026742813	6118613523
152	N-acetyltryptophan	64139700	62361762	61381822	61764226	61331156	62729169	65090071	65435653	65329279	63251417	64784465	62589917
153	Coumaric acid	58156467	57491067	55996735	52827243	55894275	56710053	56639027	56810849	56388910	62291882	60741684	60271816
154	L-Lysine	164352385	175801629	164536546	157196558	166969202	162355880	152916478	143017275	160077882	142622124	143563171	138945406
155	Phosphorylcholine	1073817139	1105653254	1014961916	1012246097	970199573	979705017	1005897325	993918355	964940998	973159167	1006979950	987417945
156	Ribose phosphate	21447400	20650521	22621830	21768248	21515865	22020631	20038348	18825978	20067781	20595545	17179505	17995912
157	Myricetin 3-glucoside	81515050	77875581	78678679	72275305	75333869	76423086	77306337	80400899	73331574	80307508	82902294	80723816
158	2'-O-Methyladenosine	16590866	17339130	15628696	18559615	18075228	18008513	17518747	18971946	18603755	17737263	17198337	17685256
159	Hexose(glucose/galactose/fructose	471500308	467578419	468603015	453733337	488691639	475158191	412863025	452176758	441433003	368333176	364925546	377007848
160	Maltol	19554218	22826969	21190593.5	20313977	22037011	20279577	20000775	18580601	15160205	17491049.5	17052953	17929146
161	Camelliaside A isomer 1	647827461	641081365	624994248	658214040	669109051	678648452	694810707	673381013	663741753	690357842	669673946	673268792
162	Ala-Ile	32000236	32849454	30217743	31663829	30558935	31984055	29056067	31741853	30579244	28436153	28446898	29247675
163	Glucosylvitexin isomer 2	100524871	96649317	94907855	100087845	105935017	105821230	123319635	125851773	117778824	125300263	126449863	129275582
164	Theogallin	2550256547	2541485607	2513714884	2493481473	2506789040	2555391054	2701084796	2736301537	2685655140	2534559883	2549209374	2609872041
165	Quercetin 3-glucosyl-rhamnosyl-galactoside	87026927	83936492	84538324	87863461	90703618	90494915	92111547	88982599	88013632	92927626	91458489	90144630
166	Quercetin 3-O-glucosylrutinoside	210222083	211605029	211995003	207276599	223553676	229316529	228537190	217460710	215199701	224334658	223530225	218161414
167	Theaflavin	304624389	299193680	289791865	269616666	274143208	287875700	274437777	272956667	265985388	277110853	282816892	285191475
168	Caffeine	91215952647	91989825368	90563865042	90983071427	92604703452	92223191527	97036850854	96490335701	95878389814	95154415286	94021674806	92417138192
169	Isoquercitrin	270297557	260887462	258340925	262173272	260607964	274861207	279264598	265682528	270382935	285276386	277964060	284843625

170	Deoxyinosine	20696126	20100370	18984461	20355253	20114166	20325652	21576109	20615575	21053390	19775617	19317709	19267194
171	Quercetin 3-(3R-glucosylrutinoside) isomer 2	489191173	472394278	473374110	479577193	502969472	503827853	517356099	492151666	478056440	504217596	490643982	497145610
172	Guanosine	172937386	176315243	169784534	202267992	205275412	197441540	193710087	201429798	193815426	178308469	174446044	178882502
173	Pro-Thr	59921965	60125720	59044028	59141659	59462743	60109524	63216288	63426939	61896997	65103509	63943955	63408091
174	Quercetin 3,4'-diglucoside	11955156	11508264	10658924	11332043	11176215	11944304	11669480	12172580	11377312	11646862	11904618	11618137
175	Digalloyl-beta-D-glucose 1	128166916	124375004	121937777	134600885	136188891	139213698	143295237	141229822	146411584	126556535	128992039	129803771
176	D-Ribose	63326187	66691099	65076135	63388906	64907164	66885206	57634412	62033775	57936575	50328205	49022602	49635538
177	Apigenin 8-C-glucoside	48260639	45886825	43823040	45598100	46946473	47134133	53248585	53151396	51159975	58723834	59107734	60206737
178	Phenylethyl primeveroside isomer	180435174	175654870	161737977	175499903	174374107	171025494	180050181	176922658	181602866	178854712	175778683	180281741
179	Abscisic acid	68250669	68233194	67585291	66680303	68476819	69063017	67811607	67722475	67884195	67738806	69466597	68820701
180	Coumaroyl quinic acid 4	566266897	563028912	549183008	537392871	546002989	558569929	552540180	548974759	551001762	565348118	579333613	575780048
181	Coumaroyl quinic acid 3	566546943	563307806	549441629	537658637	546273014	558846169	552820614	549246370	551272302	565627789	579620121	576064799
182	Trigalloylglucose	367868634	356477134	365451703	393608963	396866766	406945477	410760461	399171719	446289572	416486595	406470093	409263041
183	Threonic acid	101578372	102048914	101654182	105912571	104263501	101491668	109944754	115948662	101201091	105022219	103138205	105649337
184	Epicatechin-(4beta->8)-epicatechin-3-O-gallate isomer	35439099	34955985	34705505	32851972	30648083	31717358	33974029	37242506	37847830	34570351	34002718	33737440
185	Guanine	308409093	304996862	296576138	349835979	353272193	347199934	341785899	338294923	345687765	321303410	313493040	317679505
186	Epigallocatechin	498977701	474170183	453713299	354185994	357698990	382598990	333874850	330498091	320816093	448941783	459470353	451129033
187	L-Serine	86068010	87677130	85615019	83262784	88097799	84881297	80846958	83733915	78468399	82826168	84346334	82432003
188	Glucosylvitexin isomer 1	136027639	132580889	130500352	123251804	126915127	127373494	118470012	113423825	111783425	126265973	123258393	124581290
189	Epiafzelechin	143649566	143564353	134711932	134845700	133632142	139021493	130135545	127079299	128613704	142784228	144293230	146266066
190	Digalloyl-beta-D-glucose 2	183590483	162886095	169632340	190061725	190022850	197328593	196454170	189118268	197457345	180758624	185009331	183749065
191	Glucosylvitexin	43030393	40668036	38956185	42107279	43757346	44147919	50475545	49110835	49004964	57416229	56079925	56926330
192	Pyridoxine	56196369	57177089	51920288	54568826	55195824	56212436	60125205	55777352	55974207	63342386	62839938	61877398

193	Glu-Hyp/Hyp-Glu	95380067	93055467	90069146	90467641	94728859	96487255	98672104	98707866	97076050	93803613	94963968	92737041
194	Glycerol 3-phosphate	98649689	100143079	99991886	75703500	84287863	76087147	77195275	77531998	71185865	95052373	92929693	94533165
195	Isoquercetin isomer 2	159059209	158573018	154203493	162704622	164189923	167541736	167251420	162088868	163634736	171166809	167701077	167942215
196	NAD+	26350717	26853594	26090331	26175684	27106632	27120662	22437883	21841640	20762333	18369307	17981908	18305454
197	Isovitexin	68887629	66755861	62343994	65998055	64947600	69538941	81529480	79836427	75860072	86113249	86358309	87928668
198	Ascorbic acid	19563651	20478825	20937887	16480762	19143481	18133429	19713763	20504576	19981197	22857499	22822313	23288632
199	cis-Aconitic acid	265964264	257253543	268155726	238997630	256850542	249797292	235680485	246645465	242248471	217839629	221869078	222368487
200	L-Glutamine	852775367	924127099	861863185	859511093	862531256	880465568	742840738	755052598	736190515	663410461	659011768	673260022
201	Kaempferol-3-O-glucoside isomer	315030297	310946063	296158571	304400482	313212413	316499244	338931181	320436409	316485131	331796558	328204653	335475819
202	Benzaldehyde	14806488	14578910	12928765	15190204	13978992	13701408	14363004	14226538	14102078	14152383	13853761	13970453
203	Succinic acid	128755390	131334090	131632920	126273213	129965147	134632162	134400593	137888580	132759493	132550351	131743364	129915261
204	Acetamidobutanoic acid	34051422	37268504	39131216	32800120	31246457	31458613	37446062	39169358	32625787	53358472	52341380	53101815
205	Hydroxybenzoic acid 2	90746011	87158981	92266640	87192263	90524888	93071671	93613665	90948146	90260356	92595800	90891440	91370038
206	2-Isopropylmalic acid	168830581	172639645	170978658	158848505	173747918	177421641	170855669	174889554	169464811	172958384	176261315	174535913
207	3-O-Caffeoylquinic acid	17709402	17723365	16424431	16073014	17470211	17222471	19113702	18500977	19224172	18950014	18731696	18603402
208	Furoic acid	199794886	195250583	192310349	190009058	205045963	183118717	182920560	205765385	196158518	196400739	194158934	197686884
209	Hydroxybenzaldehyde	228823409	224107976	216208414	218493469	212187811	205512158	223003889	218048459	221439107	230220042	227378669	231360688
210	Kaempferol 3-glucosylrutinoside	1013307502	991228063	966293628	994715992	1018032782	1014188178	1051924318	1010514580	1007880089	998452240	1002910736	1015451506
211	Myricetin 3-galactoside	66712576	64979240	63586957	61559606	62454295	64938275	66076788	66363866	63160453	66853497	67928785	66974813
212	Theaflavin 3'-O-gallate	234262353	227964159	226157618	215537273	224907841	226188221	224598121	216625553	216348039	225111361	226424530	229013213
213	Traumatic acid	139665767	135694618	133446396	137482898	145352595	148549872	148906782	147712128	145160934	143345362	140872450	141902552
214	Leu-Thr	215036447	216590223	205670955	209430704	214393979	217506912	211627733	225022594	212284049	205530624	203351580	206844218
215	Benzyl primeveroside	10220559	9960007	9851810	10828993	9719807	10091437	11644886	9862364	11124561	9894097	10028557	10053692
216	Quercetin 3-O-glucuronide	6703780	6447382	6262729	6190055	6168159	6499596	7207857	6861867	6756368	6536188	6440654	6538244
217	Digalloyl-beta-D-glucose 3	282419688	267136439	268257344	287608333	295180413	300058827	340971410	297438025	307921523	283326193	287985636	284947466
218	Niacinamide	88357037	80290538	84259131	93771721	85099195	84509070	102349852	107951345	100228807	114969552	113208789	113503270

219	N-Acetyl-L-phenylalanine	29316687	28290234	27506302	27085736	27761496	28922704	30501296	29563918	30941821	29026005	29494192	29368365
220	Theobromine	1997387857	1976689957	1938119309	1993686037	1951853831	2008792330	2111688829	2154892563	2177232301	2070611575	2050878806	2037131321
221	Indole-3-carboxaldehyde	152732274	154356919	146552847	142072197	149378395	146438747	152202558	146644515	146526295	148596055	149163777	146839079
222	Quercetin diglucoside	5032583	4766382	4656772	5036811	5304036	5128342	5250554	5702831	4967681	5371117	5287059	5344336
223	Rutin	101467233	99764270	98131200	103461498	105929599	107711239	109084162	105828159	105224839	108924586	107760760	107277008
224	Coumaroyl quinic acid 1	107845246	111578636	108014550	101156436	106762029	107474186	110374833	110666388	106571605	118836245	119148492	120576269
225	L-Tryptophan	1344926886	1363041953	1327327029	1252988262	1290618603	1314267084	1312484871	1307899651	1245443408	1281919506	1286630981	1267885600
226	O-caffeoylquinic acid	29661984	29091774	29089687	27900257	29116159	30670930	29200633	28611854	29664133	30343703	30798922	30577533
227	Kaempferol-3-O-galactoside isomer	239439933	232103902	221498719	236921675	235005115	237306235	244848075	232618373	240896318	235916733	239324338	236811885
228	Isoschaftoside isomer	276633906	270592089	261360435	242075911	251927120	259651367	232560175	224215592	218734516	232021522	228632912	230706736
229	5'-Methylthioadenosine	1195095383	1179688904	1129705009	1185163708	1143404282	1160781653	1218577540	1160670301	1162872948	1228619559	1221034254	1238651060
230	Adenosine	248031145	239766113	238422145	394246021	357070278	359190472	207347073	216921558	209802514	280425400	282015107	284347486
231	Hydroxy-L-tryptophan	36335325	38825471	37066390	36583137	35371409	36339736	33253221	36165030	34876963	34249410	34375797	33915513
232	Coumaroyl quinic acid 2	12744430	12959319	12203592	12265941	13582726	12932875	12653385	12899152	12629988	12418520	12574379	12448141
233	Hydroxymethylglutaric acid	49081938	48968168	48815474	45386965	53467761	47637479	48175327	49495573	48096195	48454773	48731401	49079480
234	4-O-Methylgallic acid	1184221470	1181459186	1085787575	1206335979	1203392490	1130040859	1401540442	1355907374	1362652824	1228418029	1221257109	1212951285
235	Kaempferol	149975559	147202678	149877404	190601351	193321836	199953989	225771558	211265688	213747643	184905662	186258786	187112671
236	Epigallocatechin 3-gallate	1738127128	1755565541	1685742033	1381737958	1402763097	1450477261	1295804334	1288067141	1283690138	1721420945	1725199899	1705965100
237	Kaempferol-3-O-glucoside	542694522	523146322	517866227	514372517	546751372	559985666	578646103	556630369	561029196	555068859	561103724	560286571
238	Phenylethyl primeveroside	7022379	7015251	6750928	6552573	6436363	6681887	7180180	7338923	7991598	6750463	6810729	6736338
239	N-Alpha-acetyllysine	151359448	152481490	140477755	146237345	141607059	139932485	170486010	177148087	171795890	223597640	221185580	222183060
240	Naringenin	49855963	49155804	48044844	51214384	53146194	54008988	52892549	53674778	53623289	51895161	51395458	51869223
241	Epicatechin 3-O-gallate	1041556792	1012572568	974477727	941983601	951573317	943910014	989948599	988258307	992482769	1029857284	1023430626	1033252951
242	Pantothenic acid	43789265	44259855	43723052	42561505	44322140	45385012	44834555	44347389	44351782	42664652	43002052	43031146
243	Phloretin 2'-O-glucuronide	228290860	219153648	211104060	191943421	199693411	202575332	185981216	178622948	175984543	201618487	199940232	201457884

244	Glutaconic acid	153912972	148009878	150106057	138029297	150975361	145872502	131138850	132310305	125126440	124478102	125577694	125115264
245	Epicatechin	356497302	346079350	334549026	300997337	292833665	304498585	304560905	304898435	296544126	349132448	347090669	350043278
246	Luteolin	363390253	354405536	324445558	315557791	312506873	307718903	416751276	380285301	386030104	412513634	409181575	411343820
247	Apigenin-6-C-arabinoside-8-C-glucoside(Isoschaftoside)	472873884	459363351	434833523	468049352	475993314	485691303	563448936	562118991	540416455	589741632	591718553	587227422
248	Theaflavin-3-gallate	287819202	284848354	267509188	269475952	267733557	275996556	271409283	271883201	268645249	274632571	274507214	273041285
249	L-Malic acid	1401976817	1401040082	1400641081	1372211373	1422413199	1320789452	1361863318	1456607364	1351244091	1385836458	1385494704	1378209318
250	Epiafzelechin 3-gallate	182448945	178632154	177094850	186830010	189628650	192738799	201628069	192526531	196816739	189391625	189587668	190255025
251	L-Cystine	31597467	29895151	29603718	29622837	32967828	30473448	33173245	29105293	30903146	30812043	30718899	30667624
252	Coumaroyl quinic acid 5	15406821	14965801	14551978	14434796	14937380	15331114	14892422	14880761	14531831	14783196	14768571	14736365
253	Caffeoylshikimic acid	375890	413252	394597	365210	332883	349046.5	371042	375766	450137	400293	418666	409479.5
